# Diversity and functional traits of indigenous soil microbial flora associated with salinity and heavy metal concentrations in agricultural fields within the Indus Basin region, Pakistan

**DOI:** 10.3389/fmicb.2022.1020175

**Published:** 2022-11-07

**Authors:** Muhammad Usama Marghoob, Alejandro Rodriguez-Sanchez, Asma Imran, Fathia Mubeen, Lori Hoagland

**Affiliations:** ^1^Soil and Environmental Biotechnology Division, National Institute for Biotechnology and Genetic Engineering, Constituent College of Pakistan Institute of Engineering and Applied Sciences, Islamabad, Pakistan; ^2^Department of Horticulture and Landscape Architecture, Purdue University, West Lafayette, IN, United States

**Keywords:** salinization, metagenomics, PGP microbes, heavy metals, metabolite profiling, ACC-deaminase

## Abstract

Soil salinization and heavy metal (HM) contamination are major challenges facing agricultural systems worldwide. Determining how soil microbial communities respond to these stress factors and identifying individual phylotypes with potential to tolerate these conditions while promoting plant growth could help prevent negative impacts on crop productivity. This study used amplicon sequencing and several bioinformatic programs to characterize differences in the composition and potential functional capabilities of soil bacterial, fungal, and archaeal communities in five agricultural fields that varied in salinity and HM concentrations within the Indus basin region of Pakistan. The composition of bacteria with the potential to fix atmospheric nitrogen (N) and produce the enzyme 1-aminocyclopropane-1-carboxylic acid (*ACC*) deaminase were also determined. Microbial communities were dominated by: *Euryarchaeota* (archaea)*, Actinobacteria, Proteobacteria, Planctomycetota*, *Firimicutes, Patescibacteria* and *Acidobacteria* (bacteria), and *Ascomycota* (fungi), and all soils contained phylotypes capable of N-fixation and ACC-deaminase production. Salinity influenced bacterial, but not archaeal or fungal communities. Both salinity and HM altered the relative abundance of many phylotypes that could potentially promote or harm plant growth. These stress factors also appeared to influence the potential functional capabilities of the microbial communities, especially in their capacity to cycle phosphorous, produce siderophores, and act as symbiotrophs or pathotrophs. Results of this study confirm that farms in this region are at risk due to salinization and excessive levels of some toxic heavy metals, which could negatively impact crop and human health. Changes in soil microbial communities and their potential functional capabilities are also likely to affect several critical agroecosystem services related to nutrient cycling, pathogen suppression, and plant stress tolerance. Many potentially beneficial phylotypes were identified that appear to be salt and HM tolerant and could possibly be exploited to promote these services within this agroecosystem. Future efforts to isolate these phylotypes and determine whether they can indeed promote plant growth and/or carry out other important soil processes are recommended. At the same time, identifying ways to promote the abundance of these unique phylotypes either through modifying soil and crop management practices, or developing and applying them as inoculants, would be helpful for improving crop productivity in this region.

## Introduction

One of the biggest challenges facing agriculture in Pakistan and other arid agricultural regions worldwide is soil salinization, and models predict that this challenge is growing rapidly (Wang et al., 2020). When soils become saline, they contain excessive levels of various types of soluble salts that can stress crop plants. Adverse effects of soil salinity include reductions in soil fertility and inefficient plant metabolism, which can result in lower plant growth and reduced crop yields (Nawaz et al., 2020). The main causes of soil salinization in agricultural systems include water deficits, especially in areas with high rates of evapotranspiration, and the use of poor-quality irrigation water. One practice that is commonly used to alleviate adverse effects of soil salinity is to apply gypsum, however, this practice is not sustainable because of the high price of this amendment and negative effects that repeated applications can have on soil health over time (Nouri et al., 2017). Another way that has been suggested to try and overcome this challenge is to develop salt tolerant crops using transgenic approaches. However, this will be challenging since salt tolerance is likely controlled by quantitative traits, and it is currently difficult to correlate individual genetic elements with salt tolerance mechanisms (Ashraf and Foolad, 2013; Ben-Laouane et al., 2020). Consequently, alternative approaches to overcome the challenge of soil salinization in agricultural systems are needed (Machado and Serralheiro, 2017).

Another challenge facing agricultural systems in Pakistan and other areas of the world is the presence of elevated concentrations of heavy metals and metalloids in soil (HM). In particular, high concentrations of lead (Pb), chromium (Cr), zinc (Zn), cobalt (Co), arsenic (As), copper (Cu), nickel (Ni), cadmium (Cd), and mercury (Hg) are considered contaminants in soil (Batool et al., 2017; Rodríguez-Eugenio et al., 2018; Zea et al., 2022). High concentrations of these HM can be naturally present in some soils due to parent materials, and they can become elevated in others due to anthropogenic activities such as industrialization and mining (Shakir et al., 2016; Moryani et al., 2020; Marghoob et al., 2022). In addition to negatively impacting plant and human health, the presence of elevated concentrations of these HM can have devastating effects on critical agricultural and ecological processes in soil such as nutrient cycling and pathogen dynamics (Rodriguez-Sanchez et al., 2022). The presence of elevated concentrations of HM may be even more problematic in saline soils. For example, Cd bioavailability in soil and uptake into plants was increased in the presence of higher levels of soil salinity (Wang et al., 2014; Han J. et al., 2021), which would be highly problematic in agricultural systems growing edible crop plants.

One way to help overcome these challenges is to exploit beneficial soil microorganisms (Reeve et al., 2016). For example, some soil microbes can form intimate associations with plants, increasing their capacity to tolerate salinity and HM stress, resulting in greater crop productivity (Nawaz et al., 2021). One of the mechanisms responsible for these benefits is the capacity of some microbes to produce the enzyme, 1-aminocyclopropane-1-carboxylic acid (ACC) deaminase, which breaks down ACC, the main precursor for stress ethylene which reduces plant growth (Ben-Laouane et al., 2020). Other plant-associated microbes can promote the health and productivity of plants in high stress environments by fixing atmospheric nitrogen (N), solubilizing nutrients such as phosphorous (P), and acting as biocontrol agents against phytopathogens (Abdelrazek et al., 2020; Ben-Laouane et al., 2020; Mukhtar et al., 2020). As a result, many of these so-called “plant growth promoting” (PGP) microbes have been isolated and developed for use as inoculants to help address environmental and agricultural issues (Rilling et al., 2019).

Microbes with novel characteristics such as the capacity to promote plant growth under stress are often isolated from harsh environments. These microbes are known as extremophiles. Those with the potential to live in environments with high concentrations of soluble salts are called halophiles (Mukhtar et al., 2018). Learning more about ecological factors that influence the composition and potential functional role of halotolerant microbes with potential PGP activities could lead to the identification of practices that can help address critical agricultural challenges like salinity and HM stress. By leveraging new culture independent techniques and bioinformatics tools, it will be possible to overcome the challenge of identifying microbes that are difficult to culture and learn more about their potential functional roles in soil (Almeida and De Martinis, 2019). Therefore, the primary aim of this study was to characterize the diversity of microbial communities residing in the salt- and HM-affected agricultural lands of the Indus Basin in Pakistan. We also sought to determine how these stress factors can influence the composition of microbial communities with the potential to fix atmospheric N and produce ACC-deaminase, critical in agricultural systems. The Indus Basin is an ideal place to study these dynamics since this is an important agricultural region, it is suspected to contain soils with contrasting concentrations of soluble salts, and some fields have been reported to contain high concentrations of HM. To date, metagenomic surveys of soil microbial communities in salt- and HM-affected agricultural zones of Pakistan are scarce, and to the best of our knowledge, this will be the first report of soil microbial diversity and potential function in agricultural fields within the Mirpur Mathelo and Dhairk regions of the Indus Basin.

## Materials and methods

### Soil sampling

Soils were collected from five agricultural fields within the Indus Basin region and provinces of Punjab and Sindh, Pakistan expected to contain variable levels of soluble salts and possibly HM: Choa Saidan Shah (CSS), Chakwal, Punjab, 32.7485, 72.7574; Khewra Salt Mine Range (KSMR), Jhelum, Punjab; 32.6134, 73.0239, Pakka Anna (PA), Faisalabad, Punjab; 31.2435, 72.7998, Dhairki (D), Ghotki, Sindh; 27.945195, 69.671935, Mirpur Mathelo (MM), Ghotki, Sindh; 28.0211, 69.5490. The individual farm fields within each region were selected because they have similar crop rotations: specifically, wheat (*Triticum aestivum*) is cultivated in winter, and cotton (*Gossypium hirsutum*), maize (*Zea mays*), rice (*Oryza sativa*) and various pulse crops are cultivated in summer. Samples were collected at the land preparation stage for wheat cultivation during the months of October and November 2019. Soils were collected using a sterile soil sampling probe (2-cm width and 30.5-cm long) to a depth of 15-cm, placed in sterile containers, and transferred on ice to cold storage (4^°^C). At each location, three soil samples were collected, each consisting of multiple cores collected in a zig-zag pattern and pooled to account for heterogeneity at the site. The three fresh soil samples from each field were shipped on ice to the Microbial Physiology Lab, National Institute for Biotechnology and Genetic Engineering, Pakistan for chemical analysis, and to the Soil Microbial Ecology Lab, Purdue University, IN, United States, for DNA isolation and amplification in preparation for sequencing.

### Soil chemical characterization

Soil texture and basic chemical parameters including pH, electrical conductivity (EC), total soil organic matter (SOM), exchangeable phosphorous (P), total N, and ionic concentrations of Cl^−^ and water-soluble sodium (Na) and potassium (K) were determined using standard procedures previously described in the literature (Walkley and Black, 1934; Olsen, 1954; Richard, 1954; Imran et al., 2021; Khalid et al., 2021). Total concentrations of a wide range of elements including toxic heavy metals like Cr were quantified using a portable X-ray fluorescence analyzer (pXRF; Vanta M pXRF, with a Rh and W x-ray tube, Olympus, Waltham, Ma, United States). Briefly, 5 g of each soil were ground in a UDY grinder (3010SM/C, Seedburo, United States), packed into XRF sample cups and covered with 4.0 μm prolene film before being scanned with the pXRF. All analyses were conducted in triplicate.

### Soil DNA extraction and amplification through PCR

Total genomic DNA was extracted from each soil sample (10 g each) using DNeasy PowerSoil Kits (QIAGEN, Germantown, MD, United States) per manufacturer’s instruction. The quality and quantity of the final product was checked using a NanoDrop 2000c spectrophotometer (Thermo Scientific, Waltham, MA, United States), and stored at −20°C until use in PCR. The following primer pairs were used to amplify specific microbial taxa: 42F-376R targeting the V3-V4 region of the 16S rRNA gene for bacteria, ITS1F-ITS2 for fungi, 340F-806rB targeting the 16S rRNA gene for archaea, PolF-PolR for the *nifH* region of bacteria, and acdSF5-acdSR8 for the *acdS* gene of bacteria. PCR protocols (see Supplementary Table S1) were optimized based on previous studies conducted using the same primer sets (Op De Beeck et al., 2014; Bouffaud et al., 2018; Bahram et al., 2019; Wasimuddin et al., 2019; Yang et al., 2019). PCR products with desired band lengths were confirmed on 1% agarose gel using electrophoresis. After the first PCR, a 2nd PCR reaction was conducted to attach the CS1 and CS2 linkers in preparation for Illumina sequencing using the same conditions, but only 8 cycles. The final PCR products were stored at −20°C before shipping to the Core Genome Laboratory, Chicago, IL, United States^1^ where the products were diluted, tagged and subject to paired-end sequencing using Illumina NovaSeq.

### Screening of raw sequences

A total of 20,932,024 raw sequences were received after sequencing for bacteria, 2,381,416 for archaea, 979,543 for fungi, 15,288,910 for *nifH*, and 25,929,149 for *acdS*. To ensure that only high-quality reads were used, the sequences were subject to the following screening methods: (1) contigs assembly, (2) elimination of homopolymers, chimeric sequences and ambiguous bases, and (3) additional screening, OTU clustering, and processing of the refined data.

#### Contigs assembly

Paired-end reads were merged into contigs using mothur v 1.44 (Schloss et al., 2009). Local alignment of paired-end reads was conducted using Needleman conditions, and differences in nucleotides in the same alignment position were resolved with an ambiguous base when the difference in Phred score was 6 or lower. For the *acdS* gene, only the overlap region was selected for further analysis (Bouffaud et al., 2018).

#### Elimination of ambiguous bases, homopolymers, and chimeric sequences

Contigs with ambiguous bases or those with homopolymers >8 bp were removed. VSEARCH (Rognes et al., 2015) *de novo* identification was used to remove contigs of chimeric nature, using 1.9 as the abundance skew, 0.3 score for chimera characterization, and 0.5 as the minimum divergence ratio. In addition, a minimum of three differences was allowed when comparing segments, 8 was used as a weight of no-votes, and 1.4 was used as a pseudo-count on no-votes.

#### Additional quality screening

Non-chimeric contigs were aligned against the SiLVA SEED v138 database using Needleman conditions in both directions as an additional tool for quality screening of bacterial and archaeal 16S rRNA gene regions. Contigs having different start and end positions from expected positions of the primers were eliminated for further processing. Next, hierarchical preclustering allowing 1 error/100 bp length was used to achieve denoised contigs and generate Amplicon Sequence Variants (ASV; Huse et al., 2010; Schloss, 2020). The SiLVA NR v138 database was used as a reference to classify ASVs against specified portions of the bacterial and archaeal 16S rRNA sequences. The K-nearest neighbor algorithm and a k-mer search of 8-bp length with a cutoff of 80% were used as quality checks. Only sequences that were successfully classified at phylum or lower levels were retained for further analysis.

For quality screening of the fungal ITS region and *acdS* and *nifH* genes, hierarchical preclustering approaches allowing 1 error/100 bp length was used to denoise non-chimeric contigs into ASVs. For taxonomic screening purposes, fungal ASVs were classified using the full public UNITE database (10/05/2021) with the k-nearest neighbor algorithm and a k-mer search of 8 bp length. Contigs that were unsuccessfully classified at the phylum or lower level were eliminated.

#### OTU clustering and postprocessing of high-quality sequencing data

We obtained a total of 8,529,291 high quality reads for bacteria, 179,893 for archaea, 1,103,290 for fungi, and 36,638 and 4,408,719 for the *nifH* and *acdS* bacterial genes, respectively, after initial processing into AVSs. These high quality ASVs were subject to OTU clustering to better classify the sequences (Schloss, 2020) using the abundance-greedy clustering algorithm in VSEARCH (Westcott and Schloss, 2015). Matthew’s correlation coefficient was used to determine the accuracy of clusterization (Schloss et al., 2016). 97% similarity thresholds were used for the 16S rRNA and *acdS* genes of bacteria, 95% for the 16S rRNA gene of archaea and ITS gene of fungi, and 94% for the *nifH* bacterial gene. Elimination of singleton OTUs was performed and the most abundant sequence in each OTU was used as a representative sequence for that OTU.

Each representative sequence of bacterial and archaeal 16S rRNA genes was taxonomically classified using the SiLVA NR v138 database and the full public UNITE database (10/05/2021). For the fungal ITS region, the k-nearest neighbor algorithm and a k-mer search of 8 bp length conditions were applied. For the *acdS* and *nifH* genes, representative sequences of each OTU were classified against a selected group of sequences retrieved from the GenBank NT database. The collection of *acdS* sequences ranged from 160 to 1,500 bp in length, and for *nifH*, the collection ranged from 300 to 1,500 bp in length; both collections were retrieved from GenBank on 11/15/2021, and these databases were used to taxonomically classify each representative OTU using BLAST software (Altschul et al., 1991). A BLAST search was conducted using blastn default parameters and the best match retrieved for each sequence was considered an accurate taxonomic classification. After screening and assigning only good quality reads into OTUs on the basis of maximum similarity, there were a total of 71,737, 2,529, 1,693, 1,098 and 27,581 OTUs for bacteria, archaea, fungi and *nifH,* and *acdS* bacterial genes, respectively.

### Ecological analysis of soil microbiomes

Hill-diversity indices of order 1 (Shannon index) and order 2 (Simpson index) were used to characterize α-diversity for the marker genes used in the study (Haegeman et al., 2013). Observations were made using mothur v1.44 (Schloss et al., 2009). Pairwise comparisons among soils from the five locations were conducted by computation of ANOVA-based Tukey’s *post-hoc* test over the mean values for the Shannon and Simpson indices using the software PAST (Rodriguez-Sanchez et al., 2022). Correlations between hill-diversity indices and soil chemical parameters were investigated by calculation of Pearson’s ρ correlation computed by PAST (Rodriguez-Sanchez et al., 2022).

For β-diversity analyses, PERmutational ANalysis Of Variance (PERMANOVA; Weiss et al., 2017), Principal Coordinates Analysis (PCA; Bian et al., 2017) and cluster analysis, were used as examples of quantitative and qualitative assessments for microbial community differences among the five soil samples. For all of these analyses, the ecological information was corrected to avoid zero values by the Bayesian Multiplicative replacement method using the package *zCompositions* implemented in R, and then centered-log ratio (CLR) transformed using the package *robCompositions* implemented in R. For PERMANOVA calculation, a Aitchison distance was used. For cluster analysis, the Unweighted Pair Group Method with Arithmetic mean (UPGMA) algorithm over the Aitchison distance were used for computation.

Distinct or unique phylotypes within each of the five soils were established based on their classification as an indicator species with significant differential abundance as compared to the other soils (Hartman et al., 2018). Phylotypes were classified as an indicator for one or more soils by computation of multilevel pattern analyses based on point biserial generalized correlation coefficient using 10,000 bootstrap permutations and the package *indicspecies* implemented in R (Hartman et al., 2018). Differential abundance of a phylotype was calculated using the CLR-transformed Kruskal-Wallis tests over the abundance of each phylotype using 128 Dirichlet-Monte Carlo simulations for correction of zero values and CLR transformation through the package *ALDEx2* implemented in R.

Trophic modes and functional guilds within the fungal domain were classified using FUNGuild v1.1 with similar methodology reported before (Nguyen et al., 2016; Rodriguez-Sanchez et al., 2022). Briefly, taxonomic classification retrieved from the UNITE database (Köljalg et al., 2013) was used as input for FUNGuild v1.1. β-diversity analyses and determination of characteristic trophic modes were computed similar methods as described above.

### Correlations between dominant microbial phylotypes and soil chemical parameters

Microbial phylotypes were considered dominant in a particular soil sample if their average relative abundance for all replicates was >0.5%. Dominant phylotypes were linked to soil chemical parameters through PCA. For this purpose, the fraction of the zero-corrected, centered log-ratio transformed OTU table was used. Computations were done for individual heavy metals concentrations and other chemical parameters separately. Soil chemical parameters were previously transformed through the LOG(X + 1) equation. Similar calculations were conducted for the fungal domain classified in trophic modes.

### Functional metabolite prediction

The representative sequence for each bacterial OTU generated above was used for metagenome prediction using the PICRUSt2 methodology (Douglas et al., 2020). Briefly, representative sequences were clustered *de novo* against a phylogenetic tree containing information of 20,000 unique bacterial genomes retrieved from the IMG database (11/08/2017). Sequences were clustered on this tree using HMMER^2^ software. Next, evaluation of clustering was assessed using EPA-ng software (Barbera et al., 2019), and then GAPPA software (Czech et al., 2020) was used to provide a new tree accounting for newly-added sequences. All parameters for calculation were default. For each representative sequence, both strands were used for calculation and the best result for each was kept for analyses.

## Results

### Soil chemical properties

Details of soil texture and standard soil chemical properties are provided in Table 1. Briefly, all soils were mildly (pH 7.4–7.8) to moderately (pH 8.5–9.0) alkaline in nature, ranging from pH 7.5 in the Khewra Salt Mine Range (KSMR) to pH 8.5 in Pakka Anna (PA). EC values confirmed that the soils varied in soluble salt concentrations, increasing from the upper toward the lower course of the river. KSMR and Choa Saidan Shah (CSS) fall under the category of moderately saline (EC 4–8), PA was on the low end of very strongly saline (EC > 16), and Dhairki (D) and Mirpur Mathelo (MM) were very strongly saline, according to Abrol et al. (1988). All soils had typical characteristics of saline soils (e.g.: they were low in OM and N_,_ and had high ionic concentrations of K^+^, Na^+^, Cl^−^). Available P was below normal range for this region in CSS, while, PA and D met the recommended criteria, and KSMR and MM had higher than normal values.

**Table 1:** Texture and chemical properties of soil samples collected from five farms in the Indus Basin Region, Pakistan.

	Soil texture	pH_s_	Organic matter (%)	Nitrogen (%)	Available phosphorous (mg kg^−1^ soil)	Electrical conductivity (dS m^−1^)	Total chlorides (meq L^−1^)	Water soluble sodium (g L^−1^)	Water soluble potassium (g L^−1^)
Choa Saidan Shah (CSS)	Stony-clay	7.68 ± 0.03	1.03 ± 0.09	0.07 ± 0.01	7.31 ± 0.29	5.76 ± 0.12	2.33 ± 0.57	0.10 ± 0.02	0.02 ± 0.01
Khewra Salt Mine Range (KSMR)	Clay	7.52 ± 0.01	1.22 ± 0.12	0.06 ± 0.01	89.29 ± 4.28	6.09 ± 0.06	73.33 ± 8.02	1.66 ± 0.21	0.06 ± 0.02
Pakka Anna (PA)	Sandy loam	8.58 ± 0.04	0.65 ± 0.07	0.06 ± 0.01	31.78 ± 0.26	17.16 ± 1.17	244.00 ± 7	14.46 ± 0.48	0.22 ± 0.04
Dhairki (D)	Sandy clay	7.71 ± 0.06	1.28 ± 0.14	0.06 ± 0.01	24.79 ± 2.15	34.36 ± 1.20	2312.67 ± 19.01	91.33 ± 2.52	0.97 ± 0.10
Mirpur Mathelo (MM)	Sandy clay	8.06 ± 0.13	0.92 ± 0.09	0.05 ± 0.01	70.31 ± 4.41	32.90 ± 0.35	1063.33 ± 23.03	47.91 ± 2.73	0.50 ± 0.05

Results of the pXRF analysis and FAO safety thresholds (Rodríguez-Eugenio et al., 2018) for a select set of heavy metal and metalloid concentrations are provided in Table 2. All soils had concentrations of Ni and Co that were above safety thresholds for agricultural soils. With the exception of KSMR, all soils also had concentrations of Cr that exceeded FAO safety thresholds. Only PA had As and Cu concentrations that were above the FAO safety threshold. All soils had concentrations of Pb, Cd and Zn below safety thresholds.

**Table 2:** Concentrations of heavy metals and metalloids in soil samples collected from five farms in the Indus Basin Region, Pakistan.

	Safety threshold (FAO)	Choa Saidan Shah (CSS)	Khewra Salt Mines Range (KSMR)	Pakka Anna (PA)	Daharki (D)	Mirpur Mathelo (MM)
As	20–30	5.33 ± 0.47	7.67 ± 0.47	37.67 ± 0.47↑	18.67 ± 0.47	13 ± 1.41
Cd	01-March	n.d.	n.d	n.d.	n.d.	n.d.
Co	20–30	41.33 ± 4.71↑	34 ± 24.06↑	76.33 ± 10.4↑	89 ± 21.23↑	60.67 ± 8.99↑
Cr	20–50	84.67 ± 3.86↑	43 ± 5.35	84.33 ± 8.22↑	92.33 ± 0.94↑	63.33 ± 10.21↑
Cu	100	46.67 ± 0.94	59.33 ± 1.25	161.67 ± 1.25↑	90.67 ± 0.94	47 ± 2.16
Ni	50–100	202.67 ± 3.4↑	229.67 ± 3.86↑	628 ± 1.63↑	430.33 ± 3.3↑	462 ± 4.08↑
Pb	50–100	20.67 ± 0.94	14.33 ± 0.47	18.67 ± 0.94	26.67 ± 0.47	22 ± 1.63
Zn	300–500	41 ± 2.16	49.67 ± 1.89	60 ± 1.41	92.33 ± 1.7	73.33 ± 1.25

Values are provided in ppm (part per millions); not detected (n.d.); above threshold values:↑; Food and Agriculture organization [FAO; Rodriguez-Sanchez et al., 2022; i9183en.pdf (fao.org)].

### Soil microbial composition at the phylum level

**Figure 1:**
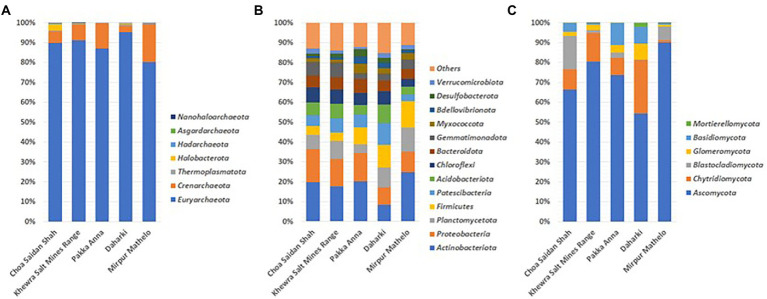
Relative abundance of dominant archaeal **(A)**, bacterial **(B)**, and fungal **(C)** phyla in five soil samples collected from agricultural fields in the Indus Basin Region, Pakistan.

The relative abundance of archaeal, bacterial and fungal communities at the phylum levels are reported in Figure 1. The dominant archaeal phylum in all soils was *Euryarchaeota* (80.10%–95.28%) followed by *Crenarchaeota* (3.28%–19.23%). Other archaeal phyla such as *Haloarchaeota Thermoplasmata* and *Hadaarchaeota* represented <3% of all samples. The dominant bacterial phyla included *Actinobacteria* (9.22%–27.15%), *Proteobacteria* (9.83%–18.44%), *Planctomycetota* (4.78%–13.29%), *Acidobacteria* (4.96%–14.71%) and *Patescibacteria* (3.52%–12.12%). Other bacterial phyla such as *Chloroflexi* or *Gemmatimonadota* represented less than 10% of the total relative abundance in all of the soils. Fungal communities were dominated by the *Ascomycota* (54.26%–89.97%) and *Chytridiomycota* (1.14%–27.03%). The relative abundance of the fungal phylum *Blastocladiomycota* was present at a substantial level (16.88%) in the CSS soil only. *Glomeromycota* and *Basidiomycota* were present at low relative abundances in all soils (1.06%–8.10% and 0.77%–11.00%).

### α- and β-diversity

**Figure 2:**
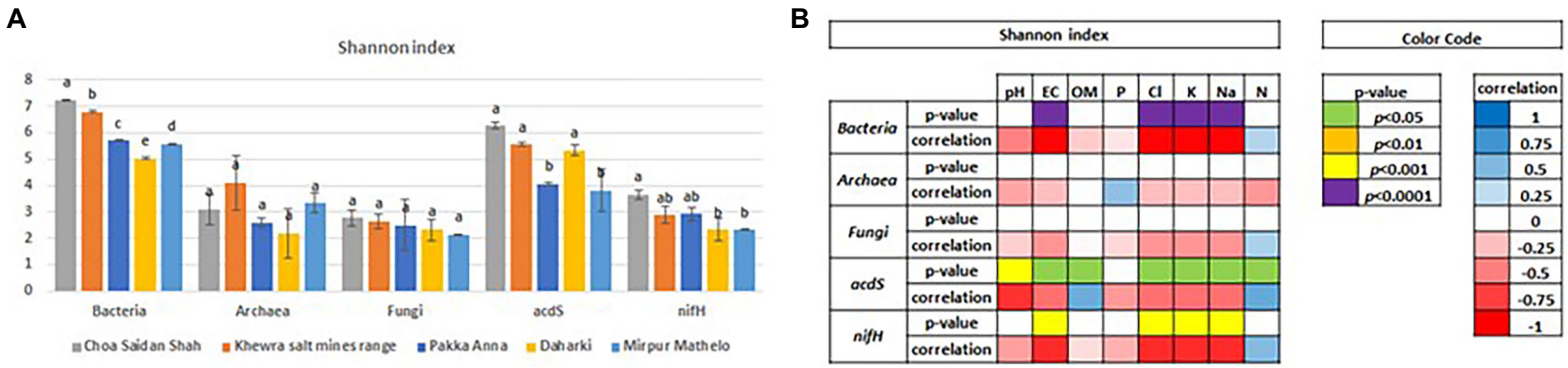
Differences in the alpha diversity of soil microbial communities in five soils collected from agricultural fields within the Indus River Basin, Pakistan, calculated using the Shannon Index (*p* < 0.05) **(A)**, and Pearson’s ρ correlations between the Shannon index and select soil chemical properties **(B)**. EC, Electric conductivity; OM, total organic matter; P, exchangeable phosphorous; CL, total concentrations of chloride; K, potassium; Na, sodium; N, nitrogen.

Results of Shannon (diversity) and Simpson (evenness) α-diversity indices are shown in Figures 2 and Supplementary Figure S1, respectively. There were no significant differences in diversity or evenness between the archaeal and fungal domains (*p* < 0.05). In contrast, there were differences in diversity and evenness within the bacterial domain and the *nifH* gene, and in the diversity of the *acdS* functional gene. In general, CSS had higher diversity for all marker genes, followed by KSMR, PA, MM and D; differences in evenness followed the same order. Pearson’s ρ correlations indicated that lower bacterial, *acdS,* and *nifH* diversity were negatively correlated, and evenness was positively correlated, with salinity parameters (EC, Cl^−^, K^+^, Na^+^).

**Figure 3:**
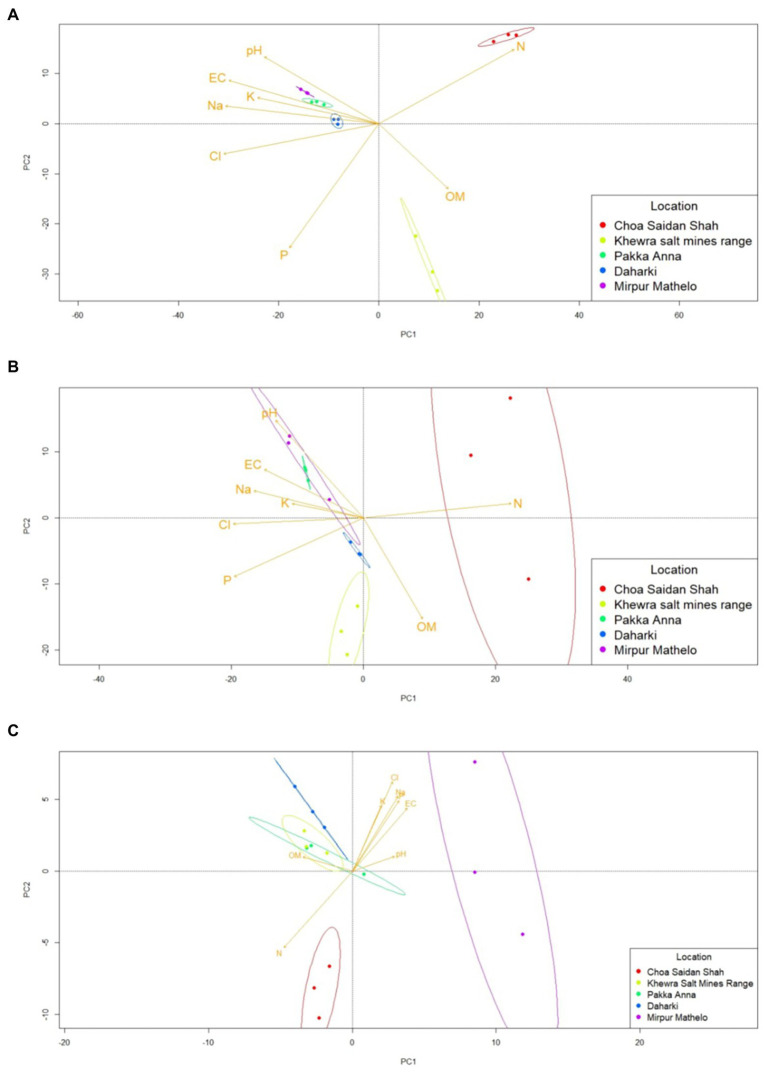
Differences in beta-diversity of soil microbial communities in five soils collected from agricultural fields within the Indus River Basin, Pakistan and correlations with chemical properties quantified using principal coordinates analyses (PCA). **(A)** bacteria; **(B)** *nifH*-containing bacteria; **(C)** *acdS*-containing bacteria; EC, electric conductivity; OM, organic matter; P, exchangeable phosphorous; CL, total concentrations of chloride; K, potassium; Na, sodium; N, nitrogen.

Results of the qualitative β-diversity analyses are shown in Figures 3 and Supplementary Figure S2, and results of the quantitative β-diversity analyses are shown in Supplementary Figure S3. CLR-transformed PCA analyses indicated that within the archaeal domain, MM and PA had distinct clusters, there was overlap between D and CSS, and no pattern for KSMR (Supplementary Figure S2). The bacterial domain clearly differentiated into three groups, with KSMR communities clustering apart from those in CSS, and communities from the other three locations clustered very closely together (Figure 3). For fungi, microbial communities within CSS were clearly separated from the rest of the samples, and fungal communities clustered with respect to trophic mode within MM clustered separately from the other soils analyzed (Supplementary Figure S2). Bacterial communities containing the *acdS* gene had a similar pattern of clustering to the bacterial domain, while those containing the *nifH* functional gene clustered in samples from CSS and MM, and communities in the other three soils overlapped (Figure 3). However, it is important to note that none of these were significantly different at the 95% confidence interval when evaluated using CLR-transformed PERMANOVA analyses (Supplementary Figure S3), so the results of the β-diversity analyses must be interpreted with caution. Cluster analyses showed similar patterns to those of PCA and PERMANOVA (Supplementary Figure S4).

### Indicator phylotypes within each soil

**Figure 4:**
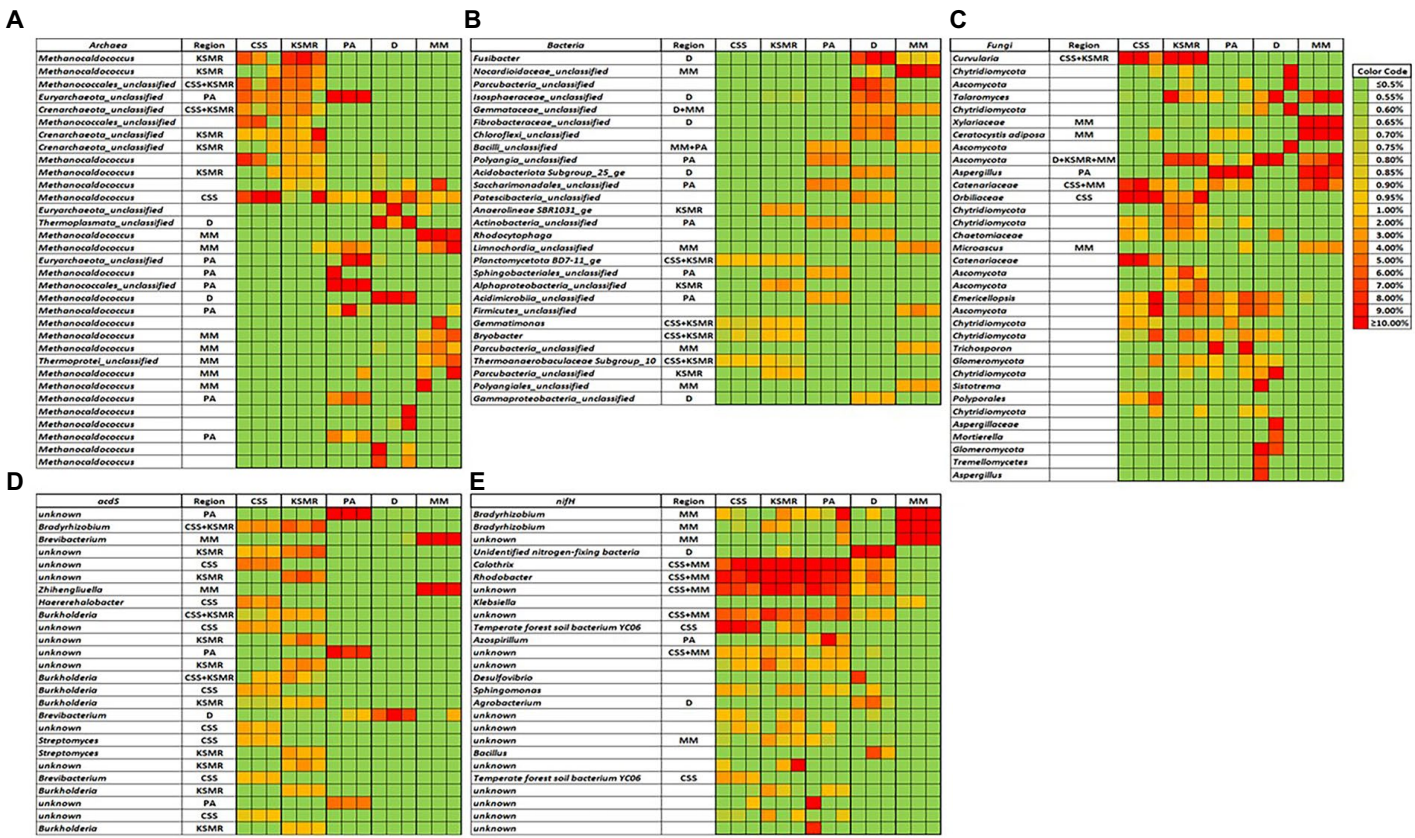
Heat maps showing the dominant soil archaeal **(A)**, bacterial **(B)**, and fungal **(C)**, phylotypes, and bacteria with *acdS* **(D)**, and *nifH* genes **(E)**, in five soils collected from agricultural fields in the Indus River Basin, Pakistan, and whether they can be considered as indicator species for a particular region. CCS, Choa Saidan Shah; KSMR, Khewra salt mines range; PA, Pakka Anna; D, Daharki; MM, Mirpur Mathelo.

Within the bacterial domain, there were many dominant phylotypes that were distinct and therefore could be considered an indicator species within the five soils (Figure 4B; Supplementary Figure S4B). For example, within CSS and KSMR, *Bryobacter* and *Gemmatimonas* were distinct; within D, *Fusibacter* and members of *Isospheraceae*, *Fibrobacteraceae* and *Acidobacteridota* were distinct; within MM, *Parcubacteria*, *Nocardiodaceae*, *Limnochordia* and *Polyangiales* phylotypes were distinct; and finally, within PA, *Saccharimonadales*, *Actinobacteria*, *Sphingobacterales* and *Acidimicrobiia* members could be considered distinct or unique for the soils. There were fewer differences among the archaeal and fungal domains (Figures 4A,C; Supplementary Figures S4A,C). For example, within the archaea, individual *Methanocaldococcus* phylotypes were distinct among the soils, however, only CSS and KSMR had *Crenarchaeota* members as unique phylotypes. For fungi, the dominant phylotype *Curvularia* was unique to CSS and KSMR, one *Aspergillus* phylotype was unique for PA, and *Ceratocystis adiposa*, *Microascus* and a *Xylariaceae* member were unique in MM. No fungal trophic modes were characteristic of any of the sites analyzed (data not shown).

Interestingly, all of the five soils had *acdS* gene-containing microorganisms that were distinct (Figure 4D). In particular, KSMR hosted several unique *Burkholderia Streptomyces*, and *Bradyrhizobium* phylotypes; *Bradyrhizobium*, *Haererehalobacter*, *Burkholderia*, *Streptomyces* and *Brevibacterium* were unique in CSS; *Brevibacterium* and *Zhihengliuella* were unique in MM; *Brevibacterium* was unique in D; and, several unknown phylogenies were unique in PA.

For the *nifH* gene, surprisingly there were no dominant phylotypes that could be considered characteristic for any of the five soils, however, CSS, KSMR and PA had several unique indicator species among the dominant OTUs such as *Calothrix* or *Rhodobacter* (Figure 4E). Two phylotypes of *Bradyrhizobium* were unique in MM, and *Azospirillum* and *Agrobacterium* were unique in PA and D, respectively.

### Correlations between dominant microbial phylotypes and soil chemical parameters

A PCA plot linking dominant bacterial phylotypes with soil chemical parameters indicated that there were three distinct clusters (Supplementary Figure S5B1). In one of them, representatives of *Fusibacter*, *Parcubacteria*, *Patescibacteria*, and *Rhodocytophaga*, among others, were positively correlated with salinity parameters (EC, K^+^, Na^+^, Cl^−^) and OM, and negatively correlated with pH. In contrast, in another cluster, representatives of *Bacilli*, *Sacharimonadales*, *Parcubacteria*, *Firmicutes*, and *Polyangiales* were negatively correlated with ion concentrations (K^+^, Na^+^,Cl^−^) and OM, and positively correlated with pH and N. Finally, in the third cluster, ion concentrations (K^+^, Na^+^, Cl^−^) and pH were positively correlated, and SOM and N were negatively correlated, with phylotypes belonging to *Gemmatimonas*, *Bryobacter*, *Parcubacteria*, *Thermoanaerobaculaceae*, and *Anaerolineae*, among others.

For the case of fungi, similar clustering of dominant phylotypes into three broad groups could be observed (Supplementary Figure S5C1). Those positively correlated with EC and SOM but negatively with pH were classified as *Mortierella*, *Aspergillus*, *Trichosporon*, *Sistotrema*, members of *Chytridiomycota*, *Glomeromycota,* and *Tremllomycetes*, among others. Those positively correlated with SOM and N and negatively correlated with EC were classified as *Talaromyces*, *Ceratocystis*, *Aspergillus* and *Microascus*. The cluster positively correlated with soil pH and EC and negatively correlated with SOM and N included members of *Curvularia*, *Ascomycota*, *Chaetomiaceae*, *Orbiliaceae* and *Chytridiomycota*, among others.

For trophic modes, two groups formed with respect to soil parameters (Supplementary Figure S5). One group was positively correlated with N and OM (trophic modes of pathotroph, symbiotroph, and pathotroph-symbiotroph), and the other were positively correlated with pH, P and concentrations of ionic concentrations of Cl, Na, and K (saprotrophs, saprotroph-symbiotroph, pathogen-saprotroph, and pathogen-saptrotroph-symbiotroph; Supplementary Figure S5F1). Pathotroph, symbiotroph, and pathotroph-symbiotroph were also positively correlated with Cu and As, whereas the other trophic modes were positively correlated with Cr, Co, Ni, Pb, and Zn (Supplementary Figure S5F2).

Within the archaea, dominant phylotypes related to *Crenarchaeota* were positively correlated with soil pH and EC, and negatively with SOM and N (Supplementary Figure S5A1).

For bacteria containing the *acdS* gene, *Zhihengliuella* was positively correlated with higher soil pH and EC, but negatively correlated with SOM and N, whereas *Bradyrhizobium*, *Burkholderia*, *Haererehalobacter* and *Streptomyces* were positively correlated with SOM and N_,_ and negatively correlated with soil pH and EC (Supplementary Figure S5D1).

Finally, for bacteria containing the *nifH* gene, *Bradyrhizobium*, *Azosporillum*, *Klebsiella* and *Desulfovibrio* were positively correlated with SOM and N and negatively with salinity and pH, while *Calothrix*, *Rhodobacter* and *Sphingomonas* were positively correlated with salinity parameters and pH, and negatively correlated with SOM and N (Supplementary Figure S5E1).

Analyses to evaluate correlations with select heavy metal and metalloid concentrations indicated that the dominant bacterial communities also separated communities into three distinct clusters (Supplementary Figure S5B2). One cluster was positively correlated with Cr, Pb, Zn, and Co, and contained *Fusibacter*, a *Parcubacteria* member, *Rhodocytophaga*, and a *Patescibacteria* member, among others. Another group was positively correlated with As, Cu, and Ni, and included *Gemmatimonas*, *Bryobacter*, and a *Parcubacteria* member, among others. Finally, the third cluster was negatively correlated with all soil metal and metalloid concentrations, and included *Polyangia* and *Parcubacteria* members, among others.

Within the archaea, many *Methanocaldococcus*-affiliated and *Crenarchaeota*-affiliated phylotypes were positively correlated with concentrations of As, Ni and Cu (Supplementary Figure S5A1). Another cluster was negatively correlated with the concentrations of all metals and metalloids, and included *Methanocaldococcus* and *Thermoprotei*.

For fungi, several phylotypes were positively correlated with all of the heavy metals and metalloids such as *Aspergillus*, *Sistotrema*, and a few *Ascomycota* (Supplementary Figure S5C1). Another cluster contained fungal members that were negatively correlated with all heavy metals and metalloids, such as *Emericellopsis* or *Trichosporon*. Several phylotypes were positively correlated with Ni, As and Cu, such as *Curvularia* or *Polyporales*. Finally, another cluster contained fungal phylotypes that were positively correlated only with Pb, Zn, Cr, and Co, such as an *Aspergillus* and *Ceratocystis adiposa*.

Bacterial phylotypes containing the *acdS* gene clustered into three distinct groups. One cluster was positively correlated with all heavy metals and metalloids and included *Zhihengliuella*, *Brevibacterium*, *Burkholderia* and *Streptomyces* (Supplementary Figure S5D1). Other phylotypes classified as *Burkholderia* and *Streptomyces* were positively correlated only with Ni, As and Cu.

Finally, bacterial phylotypes containing the *nifH* gene clustered into two groups. One was positively correlated with Ni, As and Cu, and included *Sphingomonas*, *Rhodobacter*, *Calothrix*, among others (Supplementary Figure S5E2). The other group was positively correlated with concentrations of Pb, Zn, Cr and Co, and included *Bradyrhizobium*, *Klebsiella*, *Azospirillum*, *Desulfovibrio,* and *Agrobacterium*, among others.

### Predicted metagenomes of bacterial communities

**Figure 5:**
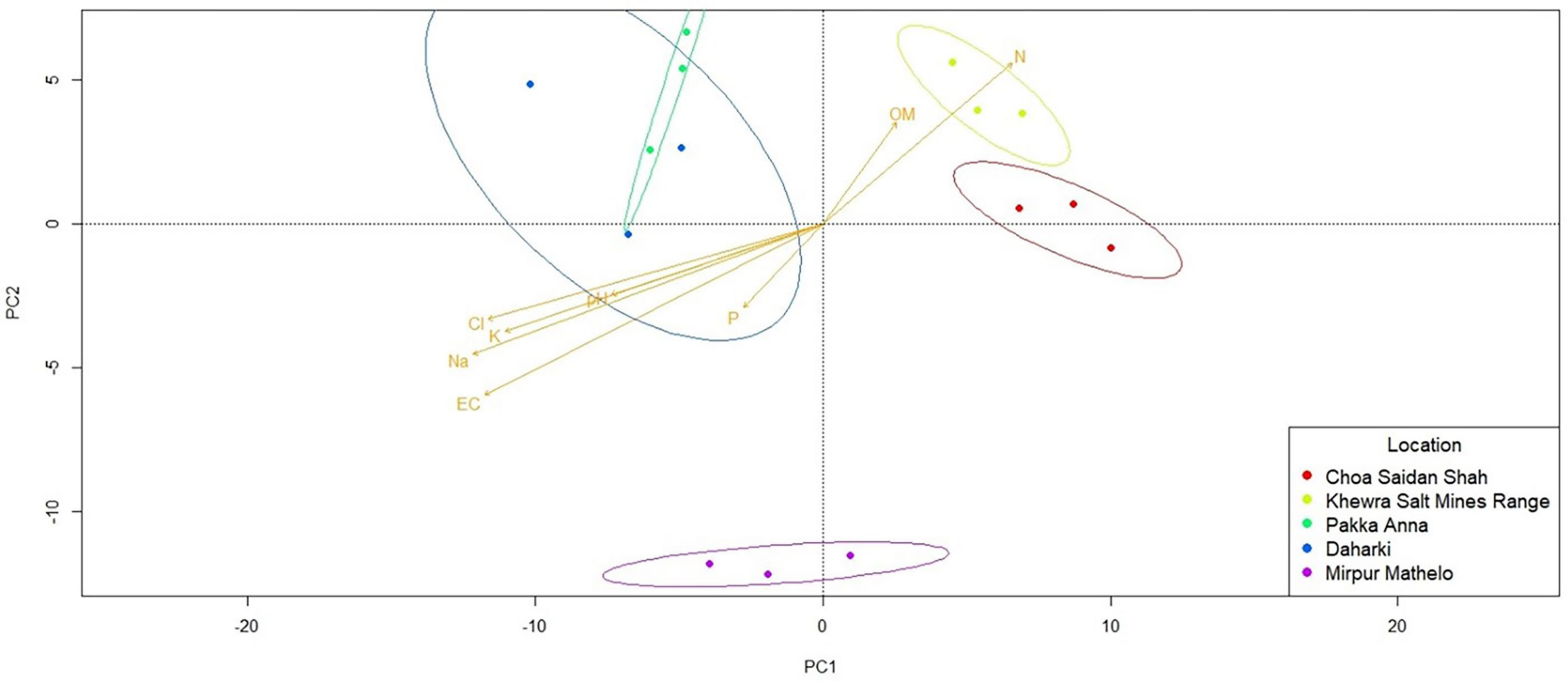
Differences in the predicted metagenome of soil microbial communities in five soils collected from agricultural fields within the Indus River Basin, Pakistan, and correlations with chemical properties quantified using principal coordinates analyses (PCA). EC, Electric conductivity; OM, total organic matter; P, exchangeable phosphorous; CL, total concentrations of chloride; K, potassium; Na, sodium; N, nitrogen.

Qualitative β-diversity metrics comparing the five agricultural soils indicated that the predicted metagenomes of the soil microbial communities clustered into 3 distinct groups: CSS and KSMR; PA and D; and MM (Figure 5). CSS and KSMR were positively correlated with OM and N, while PA and D were positively correlated with pH, P and salinity parameters. There were also significant differences in the relative abundance of some microbial genes with potential to help plants obtain nutrients and withstand abiotic stress among the five soils (Supplementary Table S2). For example, the relative abundance of microbes with genes for synthesis of ACC-deaminase (*acdS*) and nitrite reductase (*nirK*) were greater in CSS and D than the other soils. Differences in the potential for microbial communities to solubilize P were also apparent, with D having a greater relative abundance of microbes with acid phosphatase genes (*appA* and *phoN*), and CSS having a greater relative abundance of those with alkaline phosphatase genes (*phoA*, *phoB*). Microbes in D also had a greater potential relative abundance of genes for salicylate synthase (*mbtI*, *irp9*, *ybtS*), while PA had a greater relative abundance of the gene for isochorismate pyruvate lyase (*pchB*), and MM had a greater relative abundance of the gene for ferric uptake regulator (*FUR*). Finally, all soils except CSS contained microbes with a high relative abundance of the gene for indole-3-glycerol phosphate synthase (*IGPS*).

## Discussion

Learning more about how soil salinity and elevated concentrations of HM can alter the composition and functional potential of soil microbial communities has potential to help aid in the development of new practices that can help farmers overcome these challenges. As predicted, agricultural soils in the Indus River Basin in Pakistan provided an ideal location to study these dynamics. Farm fields ranged from moderately saline upstream (CSS, KSMR), to very strongly saline downstream (PA, D, MM), but did not differ in other critical soil parameters such as pH and OM (Table 1), which are generally the strongest factors driving soil microbial community structure (Rodriguez-Sanchez et al., 2022). At the same time, concentrations of Ni, Co, and Cr were all greater at sites downstream relative to upstream (Table 2). Elevated concentrations of soluble salts in soils under irrigation in arable regions are common, and likely to accumulate at higher concentrations downstream due to deposition within irrigation networks (Syed et al., 2021). The exact source of HM in this region are unclear, though it could be due to soil factors such as soil parent materials, as well effluent from salt and coal mines, nearby chemical industries, and agricultural and municipal wastes (Ashraf et al., 2021). Regardless of the cause, the combined presence of high concentrations of soluble salts and HM is of concern given that both can harm crop plants, and salinity can increase bioavailability of HM like Cd and Pb (Acosta et al., 2011). Fortunately, others have reported opposite trends for HM like Cr, Ni, and As (Han J. et al., 2021). While we cannot predict how HM will behave in these soils, we can conclude that farms in the Indus Basin are at high risk for salinity and HM, so farmers should take steps to address these stress factors.

Identifying dominant microbial taxa that thrive within these agroecosystems can provide important clues about potential threats, as well as insights on how to better manage these systems. Microbial communities in all soils were dominated by taxa with high potential capacity to withstand abiotic stress (Figure 2), which is consistent with studies in similar ecosystems. For example, Rodriguez-Sanchez et al. (2022) also found *Ascomycota* to dominate soil fungal communities in an arid agricultural region subject to salt and HM stress in Peru. Dominance of the *Ascomycota* is these systems is likely due to the fact that these microbes generally contain a high number of genes and specific traits that make them more competitive in harsh environments (Egidi et al., 2019). Members of the *Ascomycota* play important roles in soil as saprotrophs, but others can cause plant diseases (Rodriguez-Sanchez et al., 2022). *Acidobacteriota* are commonly reported as one of the most abundant groups of bacteria found within saline soils, and thus, some have proposed that they can serve as bio-indicators for salt-tolerant communities (de Leon-Lorenzana et al., 2017; Yue et al., 2020; Cao et al., 2021). Microbes within the *Acidobacteriota* are difficult to cultivate and study under laboratory conditions, so their exact ecosystem functions are still unclear, though they are expected to play important roles in biogeochemical cycles and plant growth promotion (Kalam et al., 2020). Mukhtar et al. (2018) previously found *Actinobacteria* to be highly abundant in the rhizospheres of wheat plants collected in PA. *Actinomycetes* are well-known for their capacity to tolerate conditions of high soil pH, water (Pepper et al., 2015), and salinity stress, and some have been demonstrated to help plants withstand salt stress (Djebaili et al., 2020). Many genera within the *Actinomycetes*, such as *Streptomycetes,* are also noted for their capacity to both cause as well as prevent plant disease (Schrey and Tarkka, 2008). Finally, *Euryarchaeota* are widespread in terrestrial ecosystems worldwide, including many extreme environments (Hu et al., 2013). Many of the metabolic capabilities and potential functional roles of *Euryarchaeota* in soils remain unresolved, though they are expected to play critical roles in biogeochemical cycles (Hu et al., 2013). Consequently, future efforts to isolate microbes within these taxa and elucidate their specific functional roles are recommended as a means to promote crop growth and resilience against biotic and abiotic stress.

Determining how individual taxa respond to salinity and HM concentrations is also important for learning how to better manage these agroecosystems. Like other studies (Khan et al., 2020; Wang S. et al., 2021; Chen et al., 2022; Jiang et al., 2022) salinity had a strong influence on bacterial, but not fungal diversity (Figure 3; Supplementary Figure S1) in this study. This may be related to the fact that fungi are generally more resistant to salinity than bacteria (Rath et al., 2016, 2019). Some have theorized that this could be due, at least in part, to the capacity of fungi to retain Na^+^ within their hyphae, thereby preventing translocation to more sensitive tissues (Zhao et al., 2019). Unlike salinity, elevated concentrations of HM often influence both bacterial and fungal diversity, but in different directions. For example, Stefanowicz et al. (2008) observed a decrease in bacterial and increase in fungal diversity in response to HM along a pollution transect, indicating that HM may select for fungal taxa with unique capabilities to withstand HM stress. Rodriguez-Sanchez et al. (2022) noted that higher Co and Ni concentrations in particular, appeared to be important drivers of fungal community structure in Peru. In the present study, fungal diversity did not change in response to HM, but we did identify some phylotypes such as *Aspergillus* and *Curvularia,* that were positively correlated with increasing concentrations of Co and Ni (Supplementary Figure S5C2). *Aspergillus* and *Curvularia* have been noted for their strong capacity to withstand HM stress, which could be related to their capacity to counteract HM toxicity *via* efflux pumps, biosorption, and production of secondary metabolites (Torres-Cruz et al., 2018; Imran et al., 2020). *Aspergillus* play important roles in soil as saprophytes (Nayak et al., 2020). *Curvularia* have been noted to act as pathogens in some plant species, but promote plant growth in others *via* PGP activities such as P solubilization (Priyadharsini and Muthukumar, 2017). Finally, the absence of an effect of salinity or HM on α-diversity within the archaeal community was not surprising given the strong capacity of microbes within this to tolerate extreme environments, including high salinity (Matarredona et al., 2020). Nevertheless, the fact that there were potential differences in β-diversity indicate that some phylotypes, such as some of the *Methanoncaldococcus*, could be more sensitive to these stress factors.

A diverse set of potentially bacterial phylotypes were positively correlated with increasing concentrations of soluble salts and HM in the soils downstream (Supplementary Figure S5), and thus could be targets for future isolation and/or soil management efforts. Halotolerant *Fusibacter* spp. are noted for their capacity to tolerate highly saline (Serrano et al., 2017; Selivanova et al., 2018), and HM enriched environments (Serrano et al., 2017; Meng et al., 2021; Ghosh Roy et al., 2022). *Fusibacter* spp. are thought to contribute to many important agroecosystem services including decomposition of SOM (Qiu et al., 2021), heterotrophic denitrification (Han F. et al., 2021), and arsenate/sulfate reduction (Wang and Huang, 2021). *Nocardioidaceae* have previously been found to be among the most dominant phylotypes in wheat rhizospheres grown in Islamabad and Azad Jammu and Kashmir regions of Pakistan (Latif et al., 2020), and noted for their potential PGP capabilities (Yadav et al., 2017). *Fibrobacteraceae* have also been well-documented in many wheat production systems (Prudence et al., 2021), but while they are known to efficiently utilize wheat root exudates, their exact role in plant–soil systems is not yet clear. *Limnochordia* and *Polyangiales* have been linked with organic management practices commonly used to improve soil health in saline soils (Shang et al., 2020; Yu et al., 2020). *Polyangiales* are suspected to play an important role in cellulose degradation and carbon cycles (Braga et al., 2021). Finally, *Isospheraceae* have been isolated from extreme environments (Hewelke et al., 2020; Kaushik et al., 2020; Morrow et al., 2020), but their functional role in soils is still unclear.

A number of other bacterial and archaeal phylotypes such as *Parcubacteria*, *Patescibacteria*, and *Crenarchaeota* were also positively correlated with salinity and HM (Supplementary Figures S5A,B), regardless of their association with a particular soil, indicating that they could be broadly adapted to soils in this region. *Parcubacteria* and *Patescibacteria* are members of the novel candidate phyla radiation (CPR), which tend to have small-genomes, small cell size, and limited biosynthetic capabilities relative to other bacterial groups (Moreira et al., 2021). They are suspected to act as predatory bacteria that prey on other taxa, thereby helping to keep bacterial populations in check (Nelson and Stegen, 2015; Conte et al., 2018; Mora-Ruiz et al., 2018). They have also been shown to inhabit a diverse range of environments including plant rhizospheres, and have diverse functional capabilities for carbon (C) and N cycling (Danczak et al., 2017; Selivanova et al., 2018; Nicolas et al., 2021). While these taxa have not previously been noted to be adapted to saline environment, recent studies indicate that they are influenced by HM (Huang et al., 2021; Zhang et al., 2021). *Crenarchaeota* are widely recognized as important ammonium oxidizers in soil ecosystems (Treusch et al., 2005; Nicol and Schleper, 2006; Brochier-Armanet et al., 2008). *Crenarchaeota* have been reported in saline soils (Ren et al., 2018), and soils with high concentrations of HM (Li et al., 2020), though others have found them to be negatively correlated with salinity (Oueriaghli et al., 2013). This could help explain why some *Crenarchaeota* members were classified as characteristic phylotypes in CCS and KSMR.

Several fungal phylotypes including *Mortierella*, *Sistotrema*, *Trichosporon*, and *Chaetomiaceae*, were also positively correlated with salinity parameters, regardless of their connections with individual soils (Supplementary Figure S5B). The presence of *Mortierella* spp. well documented in saline soils and several studies have indicated that they have potential to promote plant growth in these systems (Bian et al., 2020; Ozimek and Hanaka, 2020; Sun et al., 2021). Similarly, *Sistotrema spp.* (Redou et al., 2015; Grum-Grzhimaylo et al., 2018), *Trichosporon spp.* (Liu et al., 2019; Sun et al., 2021), and *Chaetomiaceae* (Castelan-Sanchez et al., 2019), have all been reported to tolerate salinity stress and have potential PGP activities. Since *Sistotrema* and *Trichosporon* were also positively correlated with SOM in the present study, future studies should consider investigating whether methods that can increase SOM, such as growing cover crops or amending soils with composts (Hoagland et al., 2008; Rudisill et al., 2015; Reeve et al., 2016; Richardville et al., 2022), can enhance their populations in these soils.

Unfortunately, not all of the phylotypes with potential PGP activities identified in this study were positively correlated with salinity and/or HM. For example, *Gemmatimonas* and *Curvularia*, were more abundant in fields located upstream where salinity and HM levels were much lower (Figure 5). Yue et al. (2020) suggested that *Gemmatimonas* can play important roles in helping plants tolerate saline conditions. *Curvularia* has also been shown to help reduce salt stress in plants (Pan et al., 2018), possibly due to the capacity of these fungi to produce osmoprotectants (Bengyella et al., 2019). However, it is important to note that some *Curvularia* species have also been reported to cause diseases in crops like wheat (Sultana et al., 2019; Bozoğlu et al., 2022; Tan et al., 2022), consequently, more focused studies on the composition and role of individual species of *Curvularia* within these soils are recommended. At the same time, identifying ways to promote beneficial populations of *Curvularia* and *Gemmatimonas* also could be helpful. For example, Li et al. (2021) observed greater populations of *Gemmatimonas* in saline soils amended with organic fertilizers, and this was linked to greater abiotic stress resistance in crop plants.

All soils contained bacterial communities harboring *nifH* and *acdS* genes, and distribution patterns suggest that individual taxa may be better adapted to different salinity and HM concentrations. For example, some *Calothrix* phylotypes were positively correlated with both salinity and HM, as well as low N (Supplementary Figure S5B1). Jiang et al. (2019) also noted positive correlations between *Calothrix* and these soil properties, and suggested that these cyanobacteria likely play an important role in suppling N in habitats with low N fertility. Others have also noted that *Sphingomonas* spp. are highly tolerant of soluble salts and HM, and can promote plant growth under these stressful conditions (Asaf et al., 2020). *Zhihengliuella* have also been positively correlated with salinity (Chen et al., 2010; Aslam and Ali, 2018; Hajiabadi et al., 2021), and shown to promote plant growth *via* their capacity to fix atmospheric N, produce ACC deaminase and IAA, and mineralize N into plant available forms (Orhan and Demirci, 2020). Many *Burkholderia* spp. have been shown to have potential PGP activities (Zhao et al., 2014; Jung et al., 2018; Sarkar et al., 2018), and promote plant growth under saline conditions (Sarkar et al., 2018). In this study, *Burkholderia* were positively correlated with HM which is consistent with other studies (Li et al., 2020), but negatively correlated with salinity (Supplementary Figure S5B). Finally, two bacterial phylotypes, *Klebsiella* and *Azospirillum*, which are well known for their PGP activity, were negatively correlated with salinity in this study (Supplementary Figure S5E2). Others have noted that *nifH* activity in isolates from these genera can be negatively correlated with increasing salinity levels (Tripathi et al., 1998), though halotolerant *Klebsiella* strains linked with high N uptake in plants grown under saline conditions have also been isolated (Sharma et al., 2016). Similarly, a salt-resistant *Klebsiella* strain isolated from a wheat rhizosphere in the Bahawalpur district of Pakistan was shown to enhance the growth of this important crop under high salinity conditions *via* the production of exopolysaccharides (EPS) and indole acetic acid (IAA), and solubilization of Zn and P (Latif et al., 2022).

Predicting metabolic profiles in metagenomic studies using bioinformatic software such as PICRUSt2 (Douglas et al., 2020) is a robust and comparatively efficient technology in the field of microbial ecology, with strong potential to help infer implications of stress factors on soil microbial functions (Kori et al., 2019). When comparing soils in study, we found that functional capabilities within D and PA were positively correlated with salinity and HM (Figure 5), indicating that microbes within these soils may have developed unique functional capabilities to deal with these stress factors. In contrast, CSS and KSMR were positively correlated with SOM and N. The reason for these differences is unclear, though since salinity (Rath and Rousk, 2015) and HM (Aponte et al., 2020) can play significant roles in activities related to SOM decomposition and other elemental cycles, it is possible that increasing concentrations of salinity and HM are altering the capacity for microbes to influence these processes. For example, Wang et al. (2018) observed a negative correlation between salinity and denitrifying bacteria harboring the *nirK* gene (Supplementary Table S2). It is unclear why soil D also was enriched in *nirK*, though it is interesting to note that this soil also had greater abundance of microbes with the gene for pyrroloquinoline-quinone synthase (*pqqC*), and the highest levels of SOM (Table 1). The *pqqC* gene plays a critical role in SOM cycling, and not surprisingly, is often linked with greater SOC and total N levels (Shi et al., 2022).

Some of the soils in the present study were also linked with several genes associated with phosphorous (P) cycling, which is consistent with other studies demonstrating that many salt-tolerant bacteria with PGP capabilities harbor P solubilizing genes (Dey et al., 2021). CSS in particular, was linked with greater relative abundance of bacteria with alkaline phosphatases (*phoA, phoB*; Supplementary Table S2), which is likely related to the very low P level in this soil. In contrast, microbes with several acid-phosphate genes (phoN, aphA, appA) were enriched in soil D (Supplementary Table S2). It is unclear why these would be enriched in this soil given that it also had high pH (Supplementary Table S2), but these findings provide further evidence that this soil is unique and deserves additional attention in future studies. Unfortunately, increasing salinity levels are often associated with decreased activity of the enzymes associated with P solubilization (Cortes-Lorenzo et al., 2012), so future efforts to isolate salt and HM-tolerant phylotypes that are capable of carrying out this critical agroecosystem activity in the presence of these extreme conditions are needed.

Interestingly, several genes associated with siderophore production were distinct in some of the soils (Supplementary Table S2). For example, microbes with the ferric uptake regulator (*FUR*) gene were enriched in MM. The capacity for bacteria to leverage this approach to sequester iron under oxidative stress is an important adaptive trait in stressful environments (Duangurai et al., 2018; Wang H. et al., 2021). Microbes with genes associated with salicylate synthetase (*mbtI, Irp9*, *ybtS*) were enriched in D, and *pchB*, which is involved in salicylic acid synthesis, was enriched in PA (Supplementary Table S2). The capacity for microbes to synthesize secondary metabolites involved in salicylic acid production is linked with ferric-chelating siderophores, and can play a critical role in help microbes withstand biotic and abiotic stress (Gehring et al., 1998; Pelludat et al., 2003; Shelton and Lamb, 2018; Mishra and Baek, 2021). In addition, bacterial production of these compounds can help promote plant growth under iron-deficient environments (Mishra and Baek, 2021), and is thought to play a critical role in helping prevent plant disease (Köhl et al., 2019). Consequently, future efforts to isolate salt and HM-tolerant microbes with the capacity to produce these compounds could be very helpful in developing future inoculants to promote crop growth.

Finally, the capacity for microbes to thrive in stressful environments and promote plant growth will require isolation of microbes with high stress tolerance. Interestingly, all soils except CSS were enriched in bacteria with the gene for indole-3-glycerol phosphate synthase (*IGPS*), which is associated with tryptophan-independent indole acetic acid (IAA) production (Supplementary Table S2). Upregulation of IGPS under salinity stress has been observed in many studies (Ahmad et al., 2020; Rangseekaew et al., 2022), and is thought to be important in helping bacteria tolerate these extreme conditions. IAA is also an important plant hormone regulating root growth, and bacterial production of this compound has long been associated with potential PGP activity (Glick, 2012). Similarly, ACC-deaminase production, which is controlled by the gene *acdS*, is a critical PGP trait. D had a greater potential relative abundance of microbes with this gene (Supplementary Table S2), so again, this soil appears to be a particularly valuable soil for future efforts to isolate soil bacteria with PGP capabilities.

Unfortunately, using the FUNGuild tool (Nguyen et al., 2016), we noted a negative correlation between salinity and some HM on fungi with symbiotrophic activity, and positive correlation between pathotrophic fungi and N and some HM within the soils of this region (Supplementary Figure S5F1). Relationships between fungi with these trophic modes and soil chemical properties have been mixed in other studies (Lin et al., 2022; Zhu et al., 2022), indicating that other site-specific factors are likely to play a role in mediating these dynamics and further research is needed to elucidate the mechanisms responsible. Fortunately, fungi with saprotrophic capabilities were positively correlated with salinity and some metals, providing further evidence of the adaptability of this important group of fungi in these soils.

## Conclusion

Agricultural fields within the Indus Basin are clearly at risk for salinity and HM stress, and farmers need new strategies to protect their crops and reduce human health risks. Results of this study demonstrate that several farm fields within this region contain indigenous microbial flora with unique capabilities to survive under these harsh conditions, and some may also be able to help promote plant growth. Overall, fields in location D appear to contain microbes with the most unique capabilities, and thus would provide a good starting point to begin to try and isolate unique taxa for future development as PGP inoculants. At the same time, we noted the dominance of microbes that could be a potential threat in these systems, as well as situations where microbes with well-known PGP capabilities were negatively impacted by salt and HM concentrations. Thus, future efforts to isolate and determine the exact functional roles of these microbes is recommended, along with strategies on how to better promote or prevent their abundance using different soil and crop management practices.

## Data availability statement

The data presented in the study are deposited in the GenBank SRA repository, accession number PRJNA869745.

## Author contributions

MM: conceptualization, funding acquisition, data curation, formal analysis, software, investigation, writing—original draft, and review and editing. AR-S: conceptualization, data curation, formal analysis, software, validation, writing—original draft, and review and editing. AI: conceptualization, supervision, and review and editing. FM: conceptualization, supervision, validation, and review and editing. LH: conceptualization, funding acquisition, project administration, supervision, validation, and review and editing. All authors contributed to the article and approved the submitted version.

## Funding

This work was supported by the International Research Support Initiative Program (IRSIP) fellowship to Ph.D. scholar MM, funded by Higher Education Commission (HEC), Pakistan IRSIP Fellowship No. (PIN) IRSIP 43, BMS 97.

## Conflict of interest

The authors declare that the research was conducted in the absence of any commercial or financial relationships that could be construed as a potential conflict of interest.

## Publisher’s note

All claims expressed in this article are solely those of the authors and do not necessarily represent those of their affiliated organizations, or those of the publisher, the editors and the reviewers. Any product that may be evaluated in this article, or claim that may be made by its manufacturer, is not guaranteed or endorsed by the publisher.

## Footnotes

^1^ https://rrc.uic.edu/cores/genome-research-core/

^2^ www.hmmer.org
