# Supplementary material for: Diversity and functional traits of indigenous soil microbial flora associated with salinity and heavy metal concentrations in agricultural fields within the Indus Basin region, Pakistan
Source: Front Microbiol. 2022 Nov 7;13:1020175. doi: 10.3389/fmicb.2022.1020175 (PMC9676371; doi:10.3389/fmicb.2022.1020175)
Supplement: Supplementary file 1 [file Data_Sheet_1.docx]

**
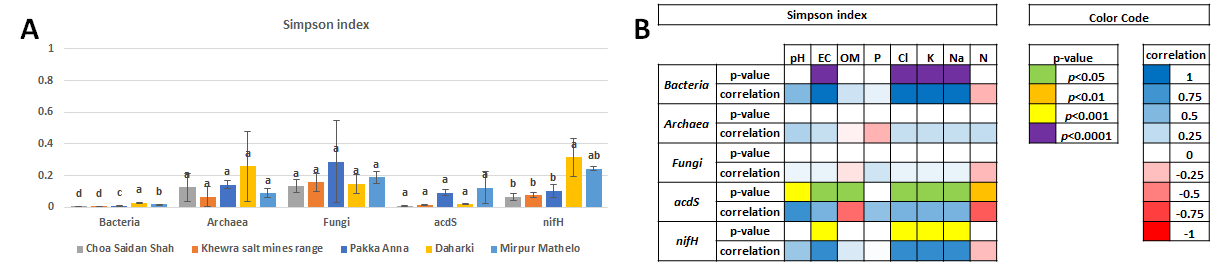
**

**Figure S1.** Differences in the alpha diversity of soil microbial communities in five agricultural soils within the Indus River Basin, Pakistan calculated using the Simpson Index (p<0.05) **(A)**, and Pearson’s ρ correlations between the Shannon index and select soil chemical properties ***(B)****. Electric conductivity (EC); total organic matter (OM); exchangeable phosphorous (P), and total concentrations of chloride (Cl), potassium (K): sodium (Na), and nitrogen (N).*


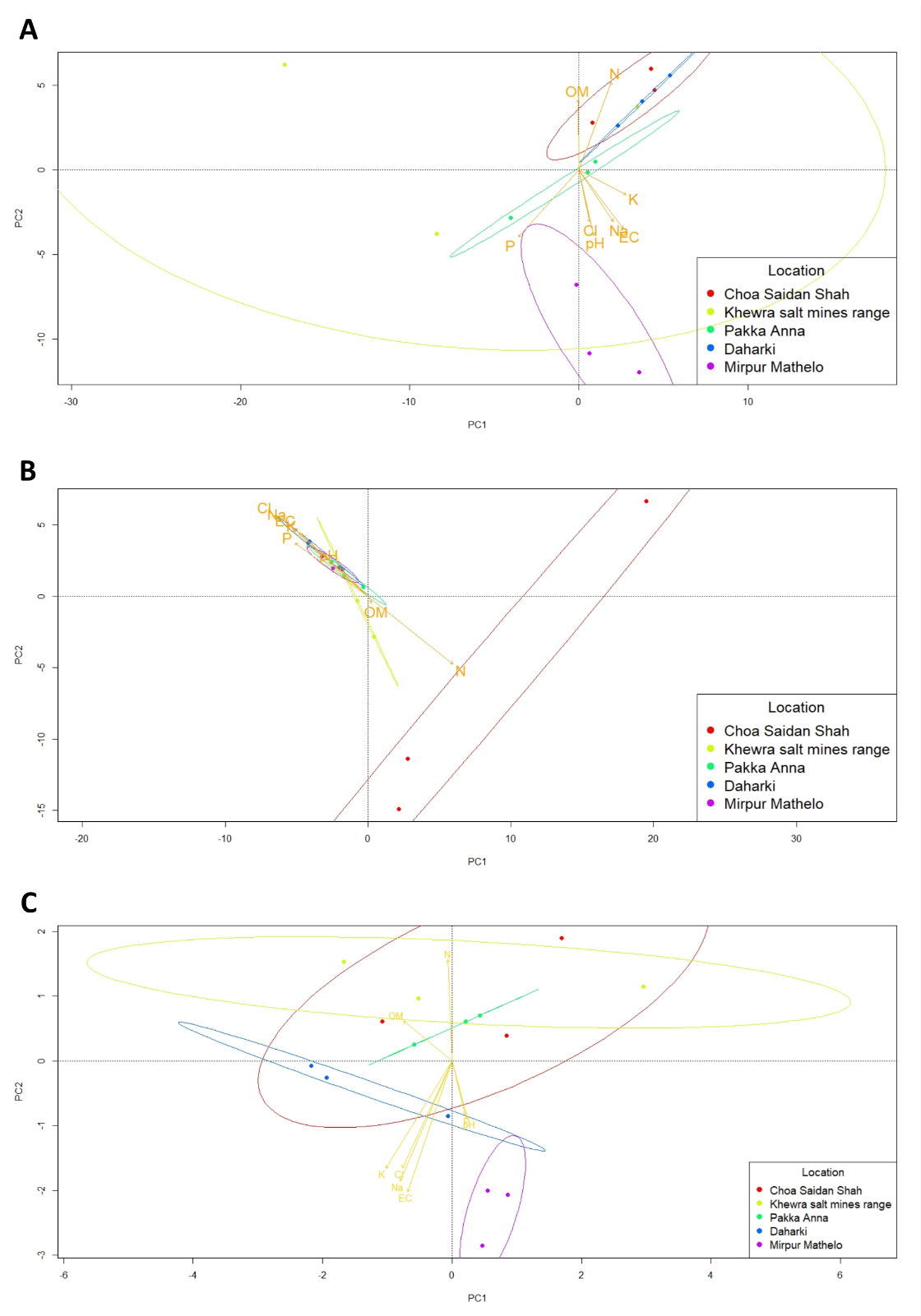


**Figure S2.** Differences in beta-diversity of soil archaeal (A) and fungal communities at phylotype (B) and functional guild (C) levels in five soils collected from agricultural fields within the Indus River Basin, Pakistan and correlations with chemical properties quantified using principal coordinates analyses (PCA). E*lectric conductivity (EC); organic matter (OM); exchangeable phosphorous (P), and total concentrations of chloride (Cl), potassium (K): sodium (Na), and nitrogen (N).*

**Figure S3.** Pairwise PERMANOVA results for the soils according to the marker genes used in the study. A: Archaea; B: Bacteria; C: Fungi; D: *acdS*; E: *nifH*; F: fungal trophic modes; PA: Pakka Anna; KSMR: Khewra Salt Mine Range; CSS: Choa Saidan Shah; D: Daharki; MM: Mirpur Mathelo. Colored cells indicate p-value <0.1 and >0.9, white cells indicate p-value >0.1.

Figure S4 - Cluster analyses showing differences among soil samples in this study. A: Archaea; B: Bacteria; C: Fungi; D: *acdS*; E: *nifH*; PA: Pakka Anna; KSMR: Khewra Salt Mine Range; CSS: Choa Saidan Shah; D: Daharki; MM: Mirpur Mathelo. 1, 2, 3 indicates the replicate number for each sample.


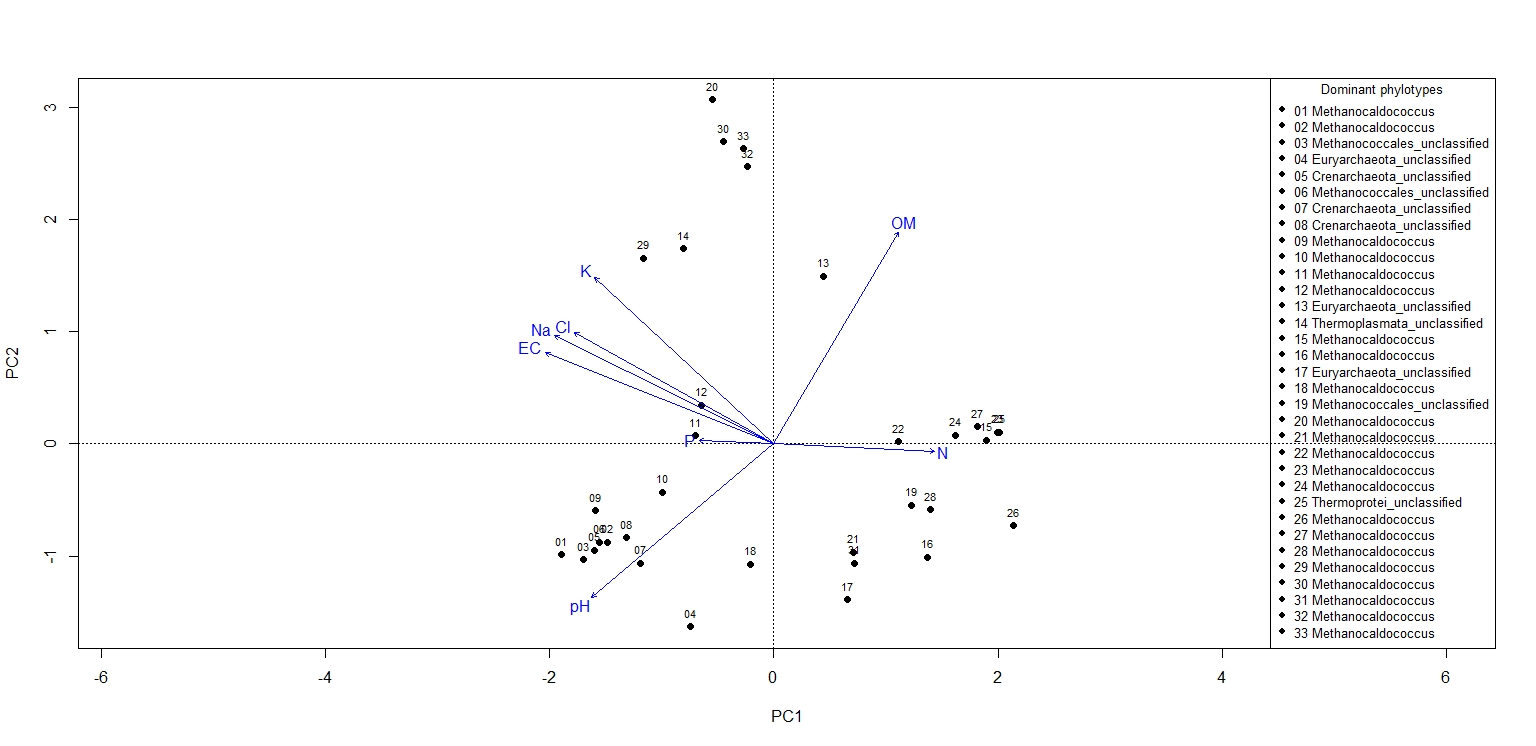


A1


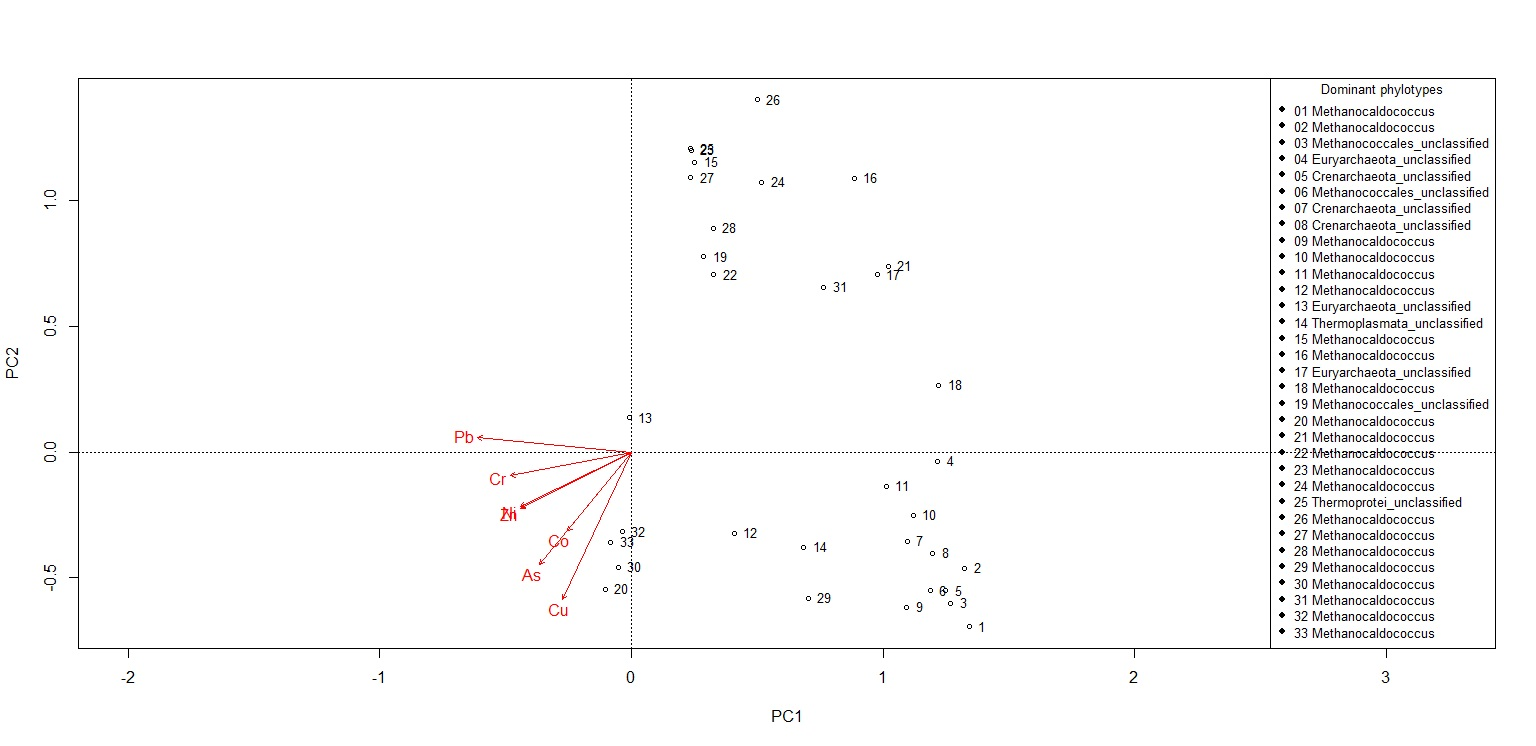


A2


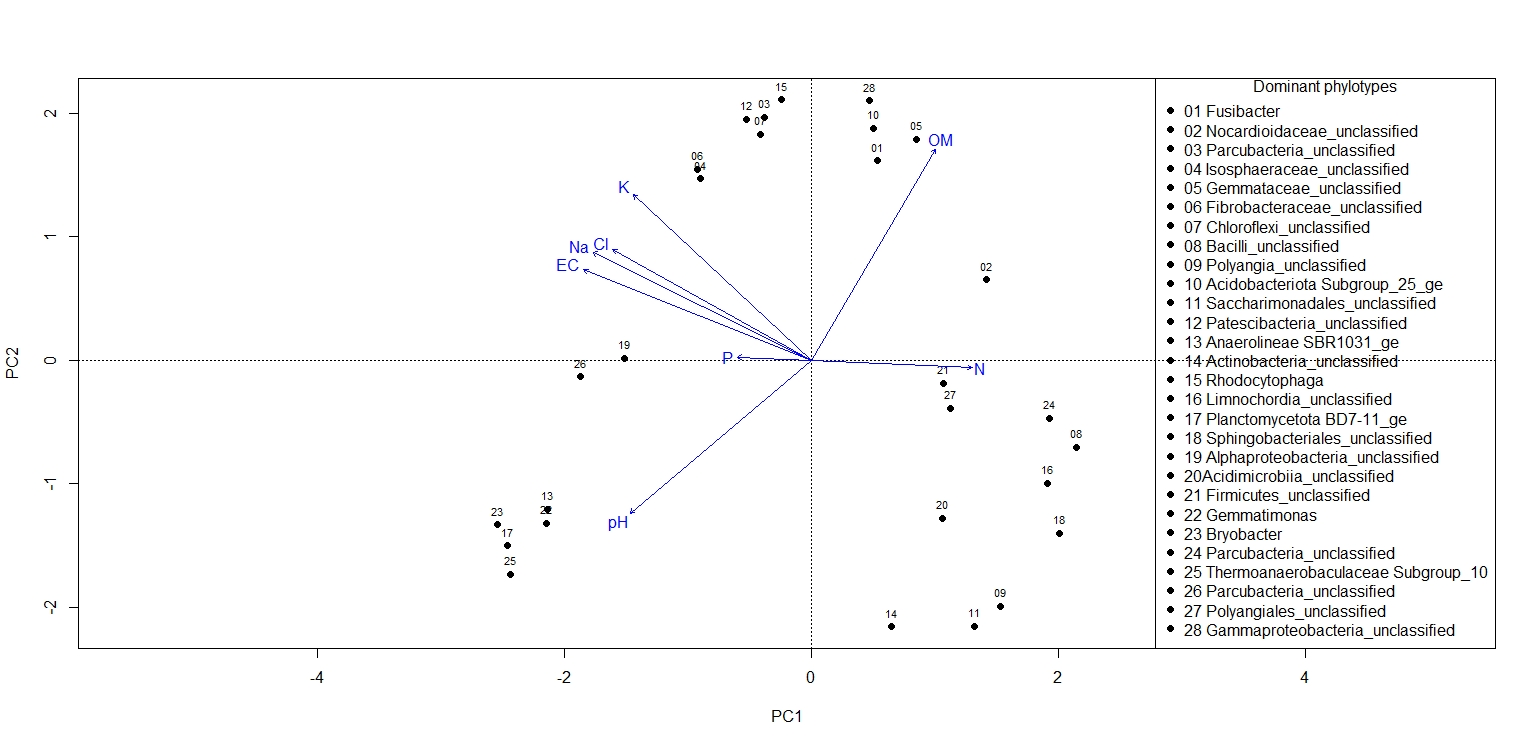

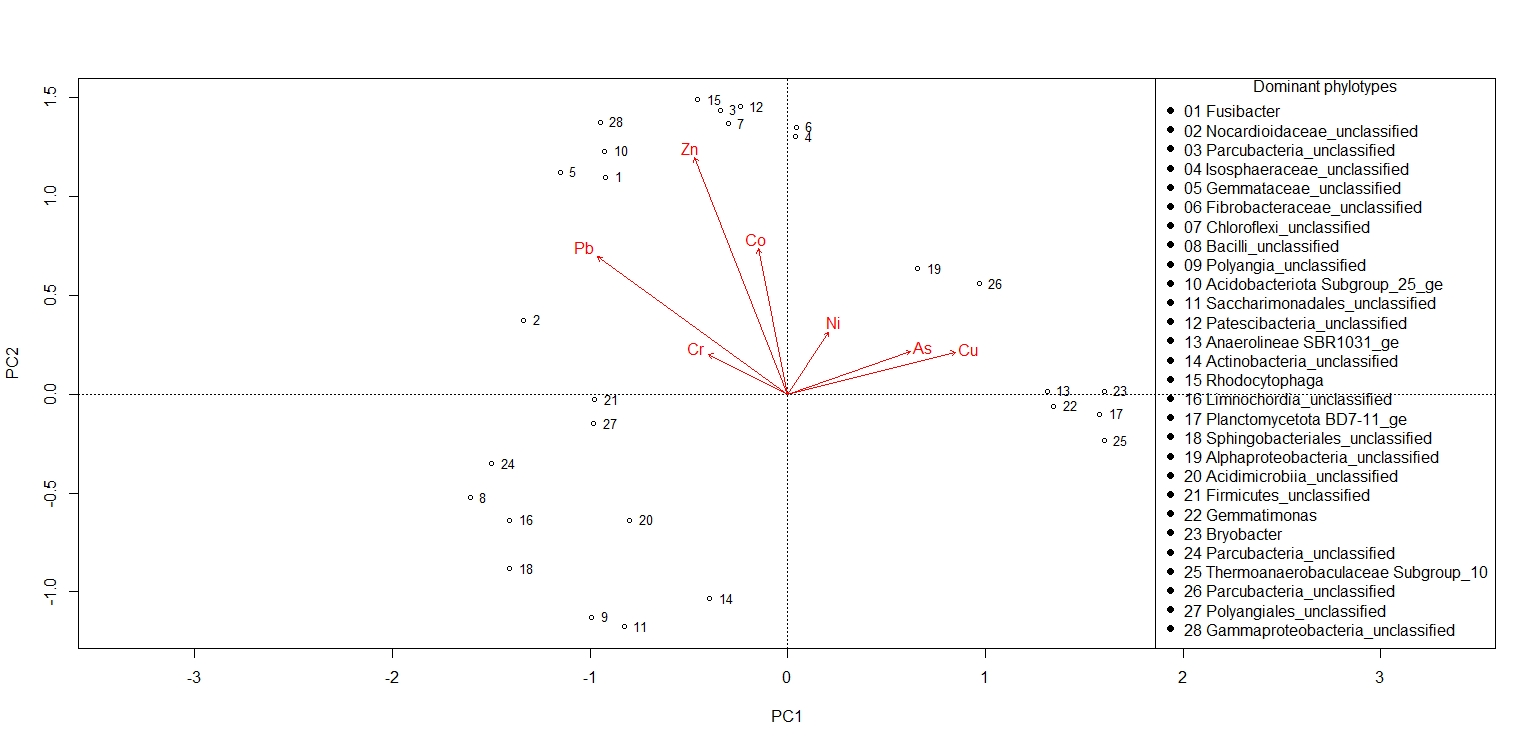


B2

B1


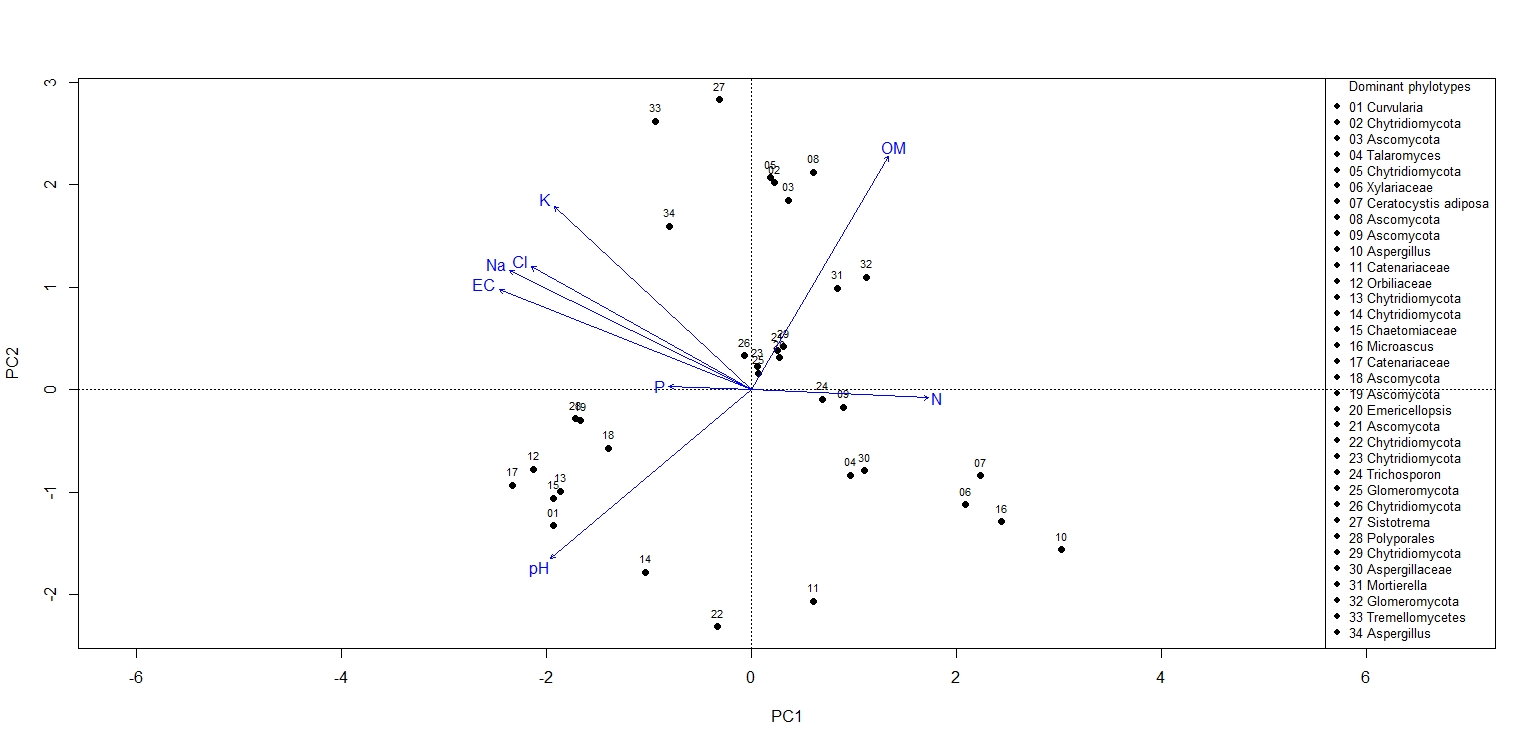


C1


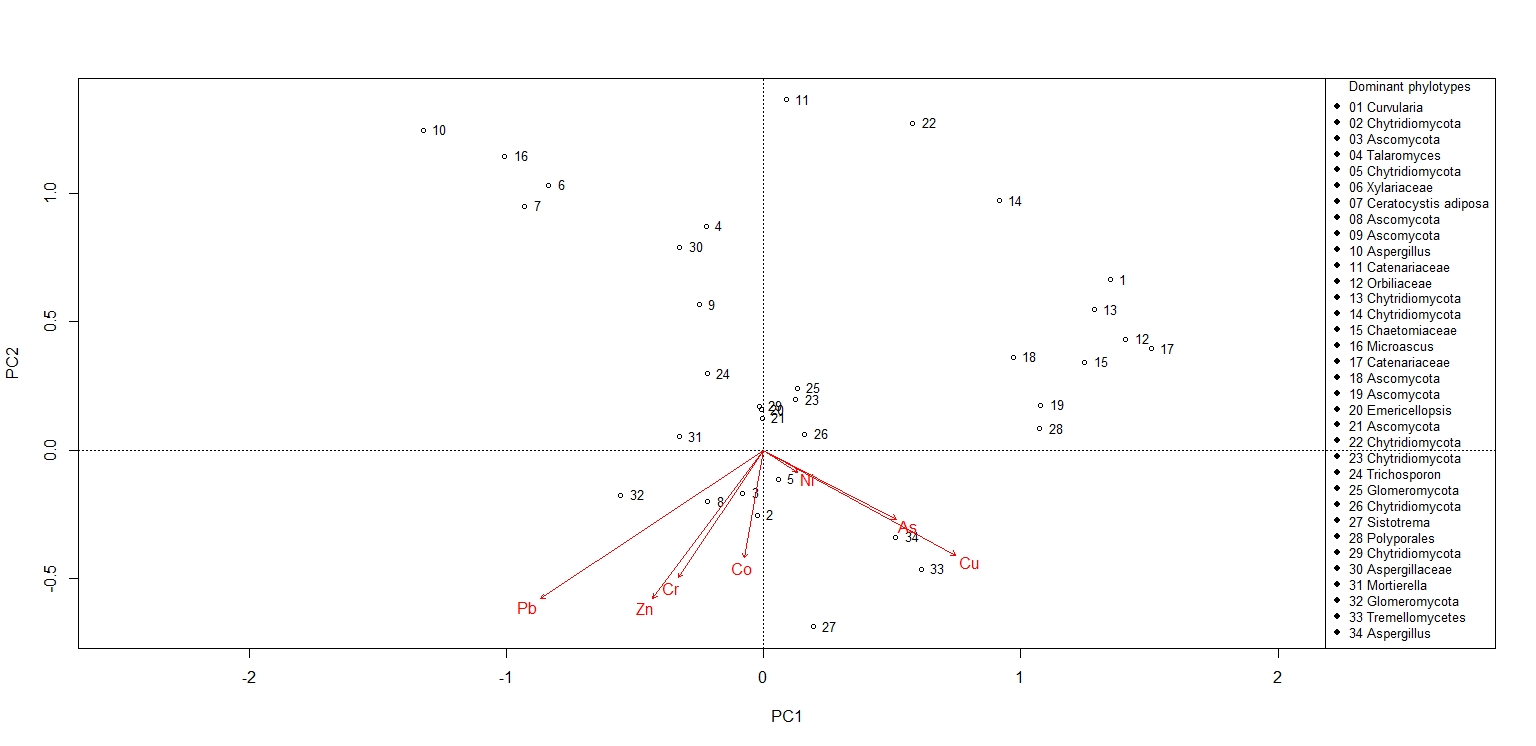


C2


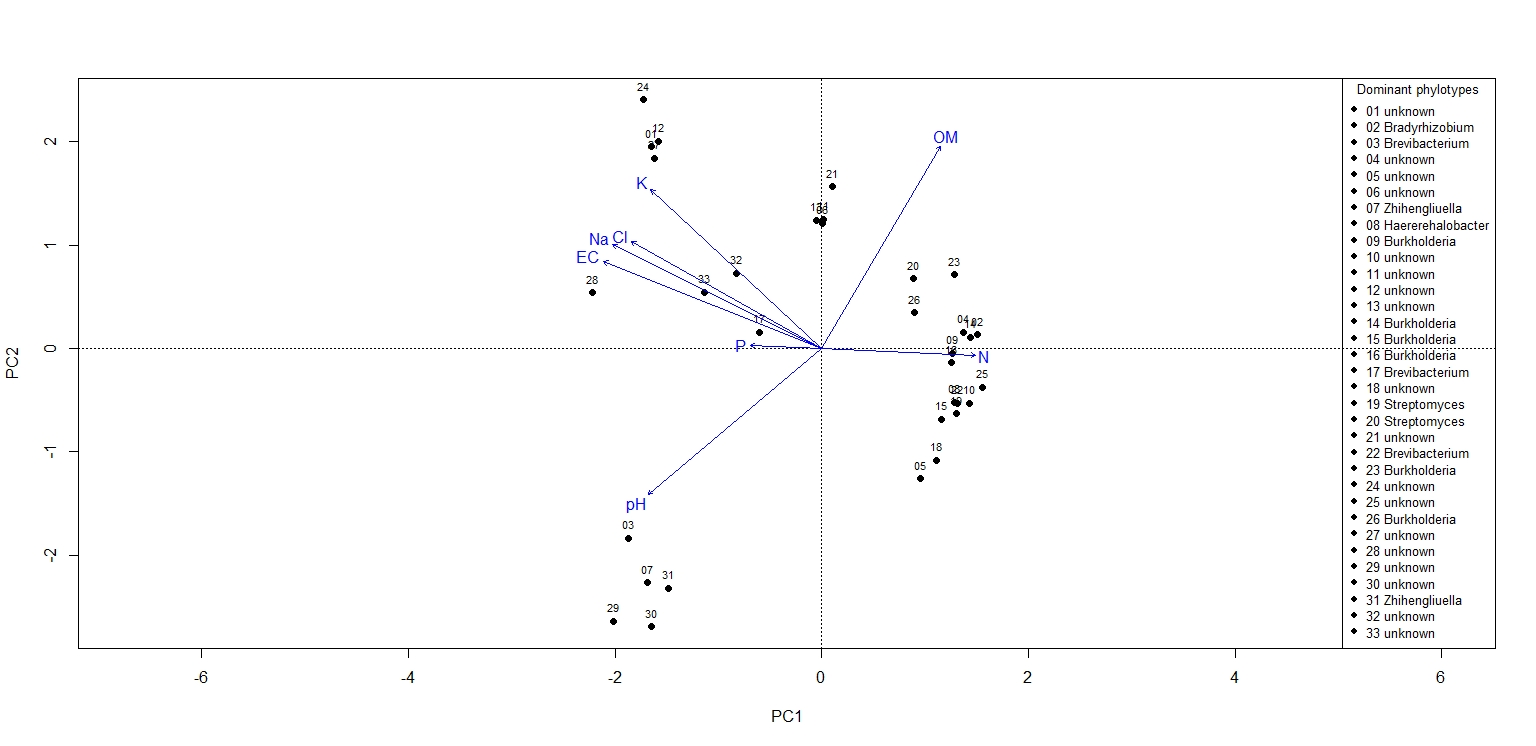


D1


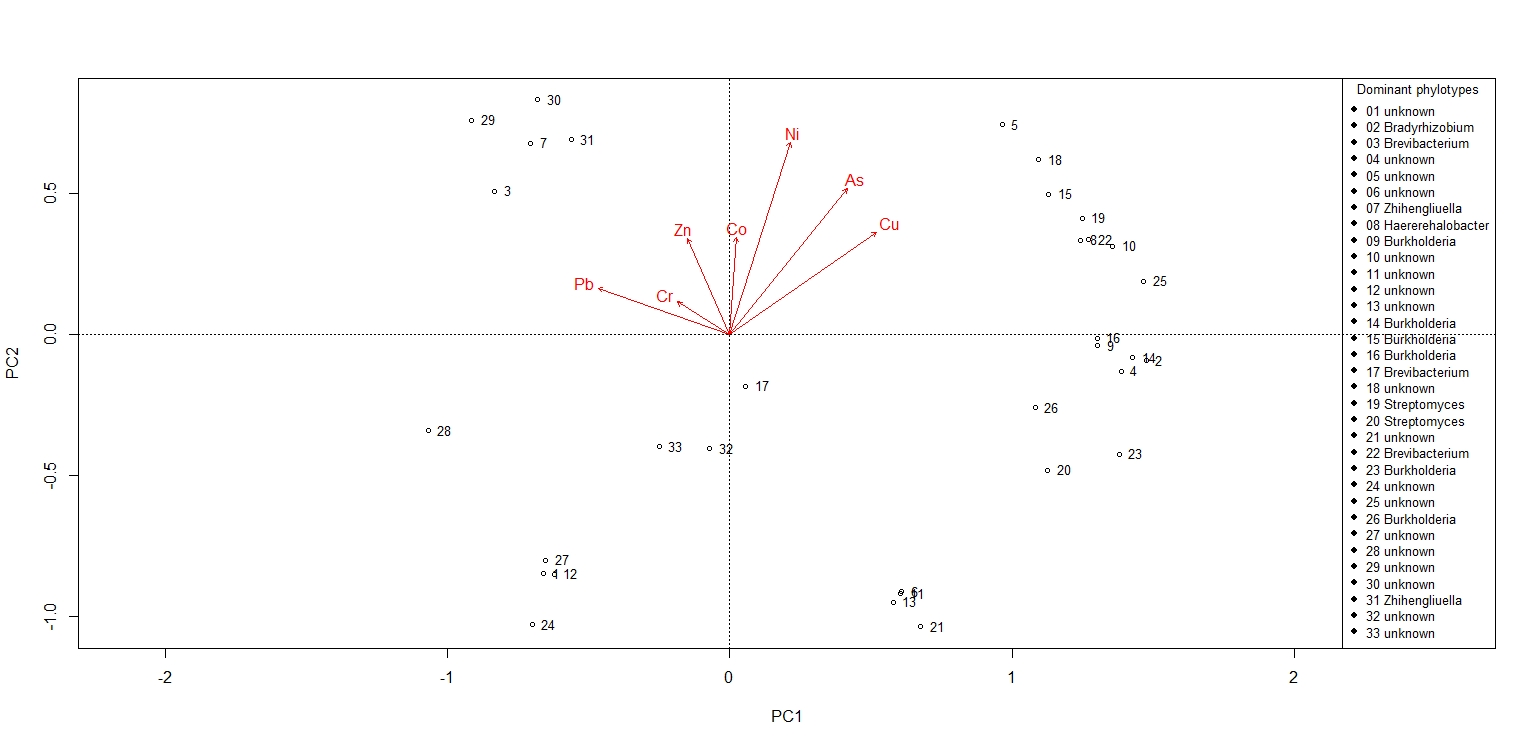


D2


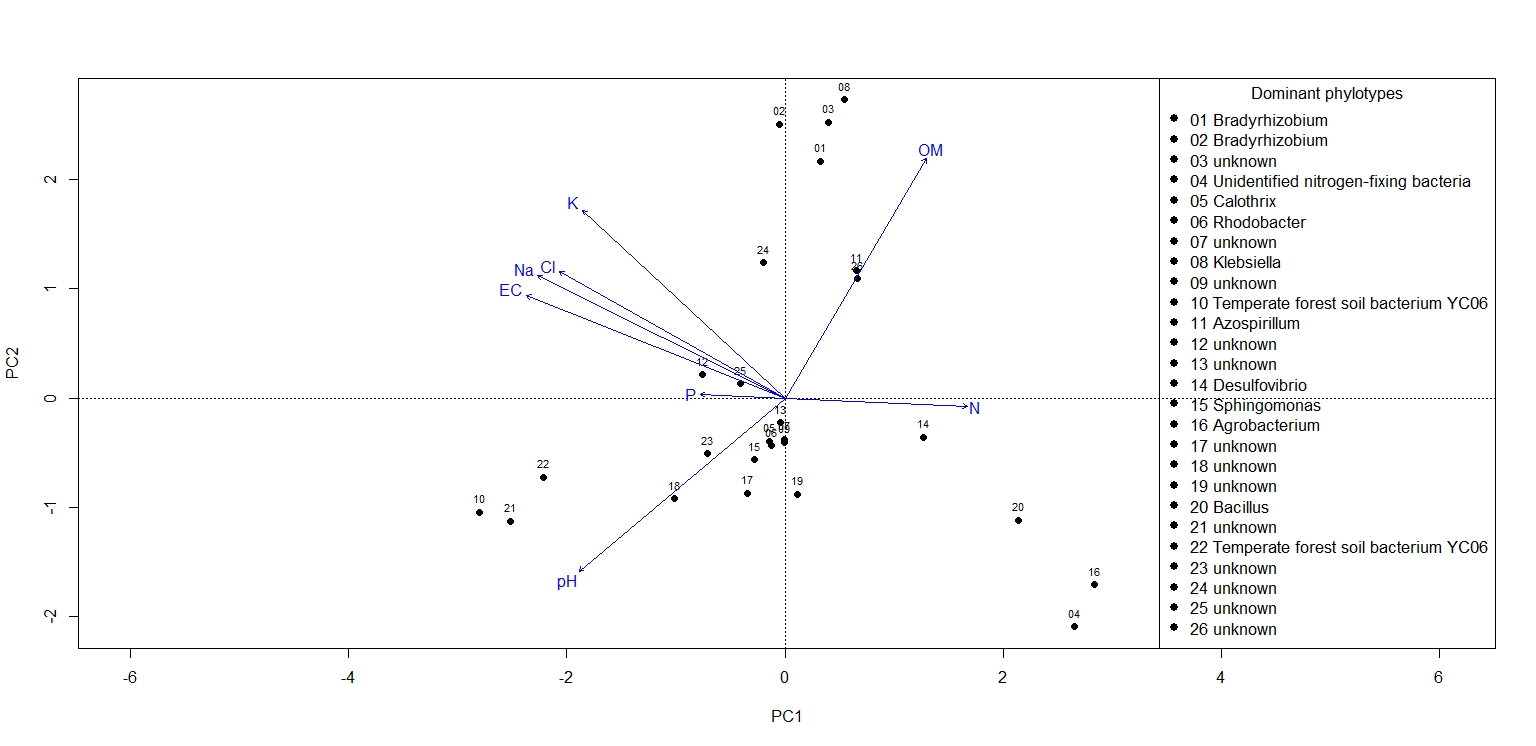


E1


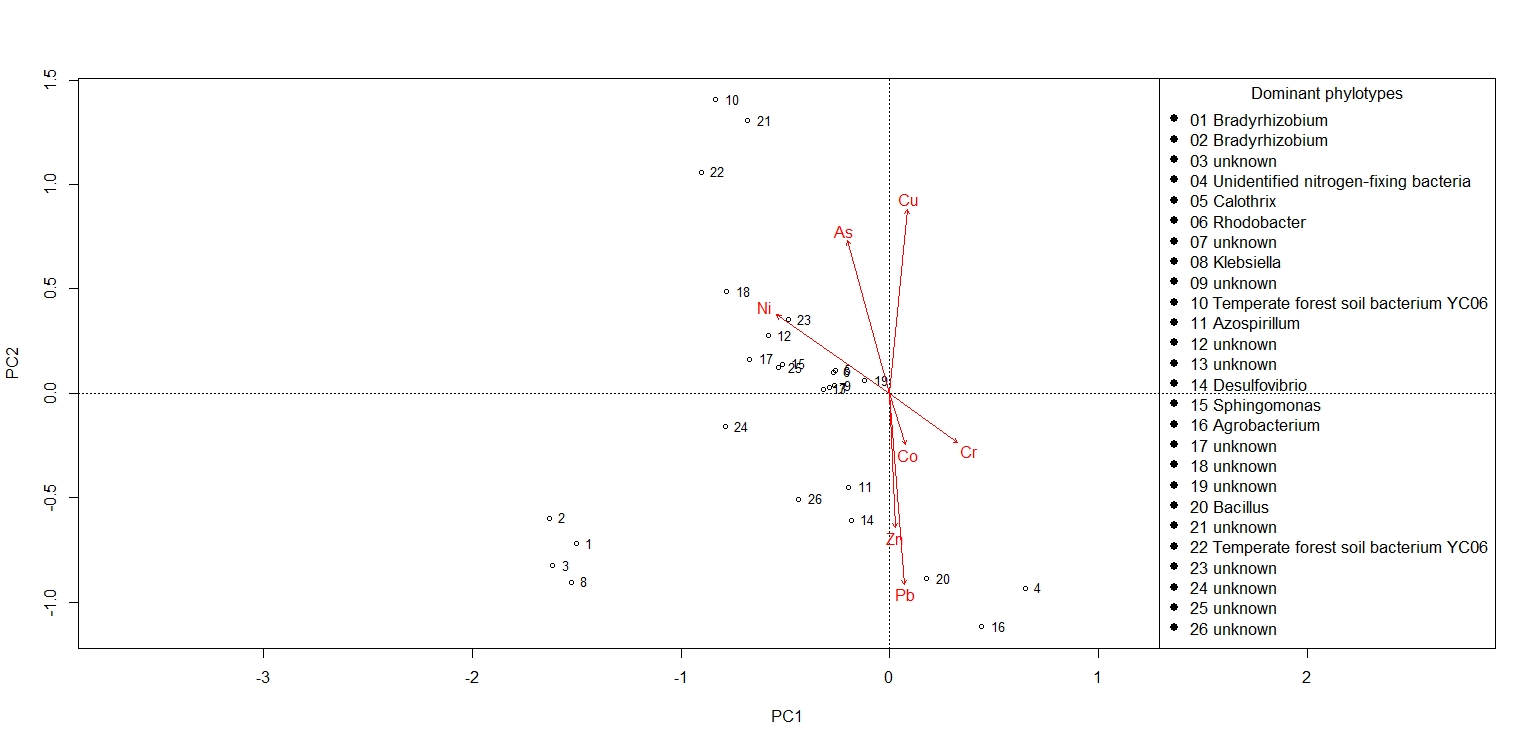


E2

**
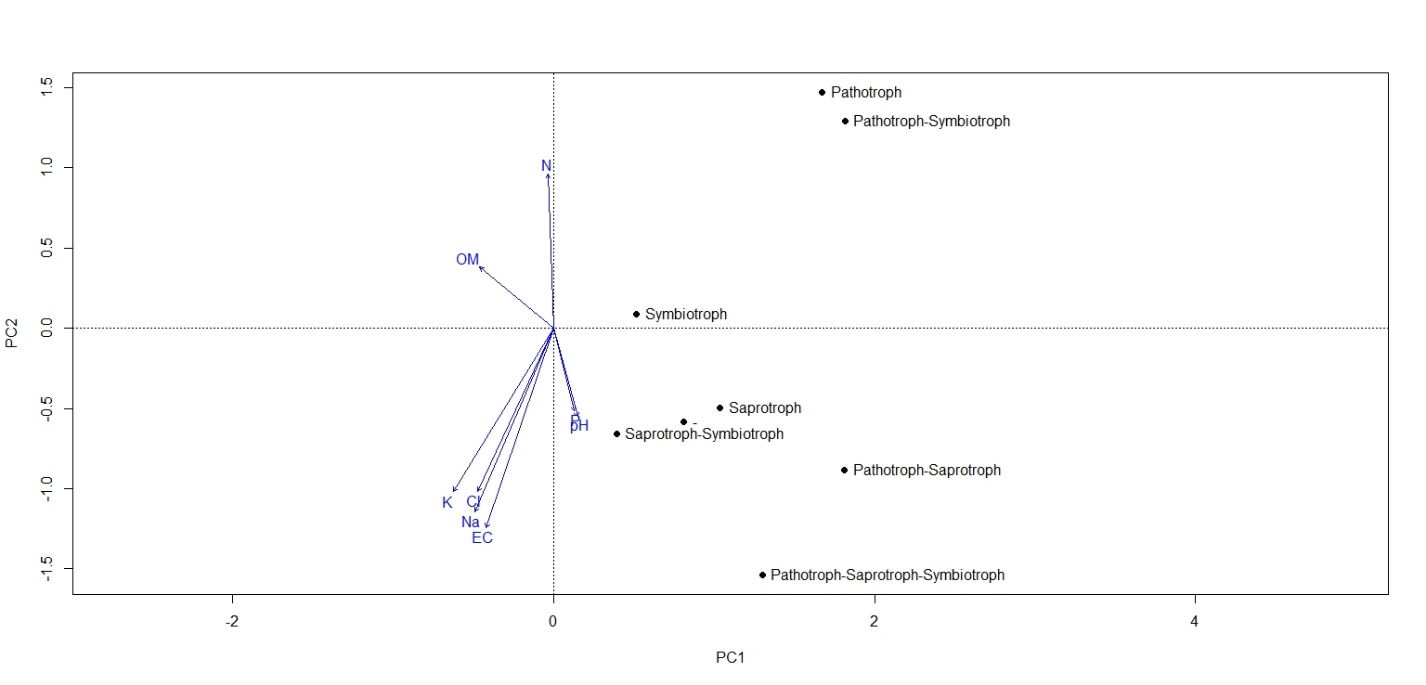
**

F1

**
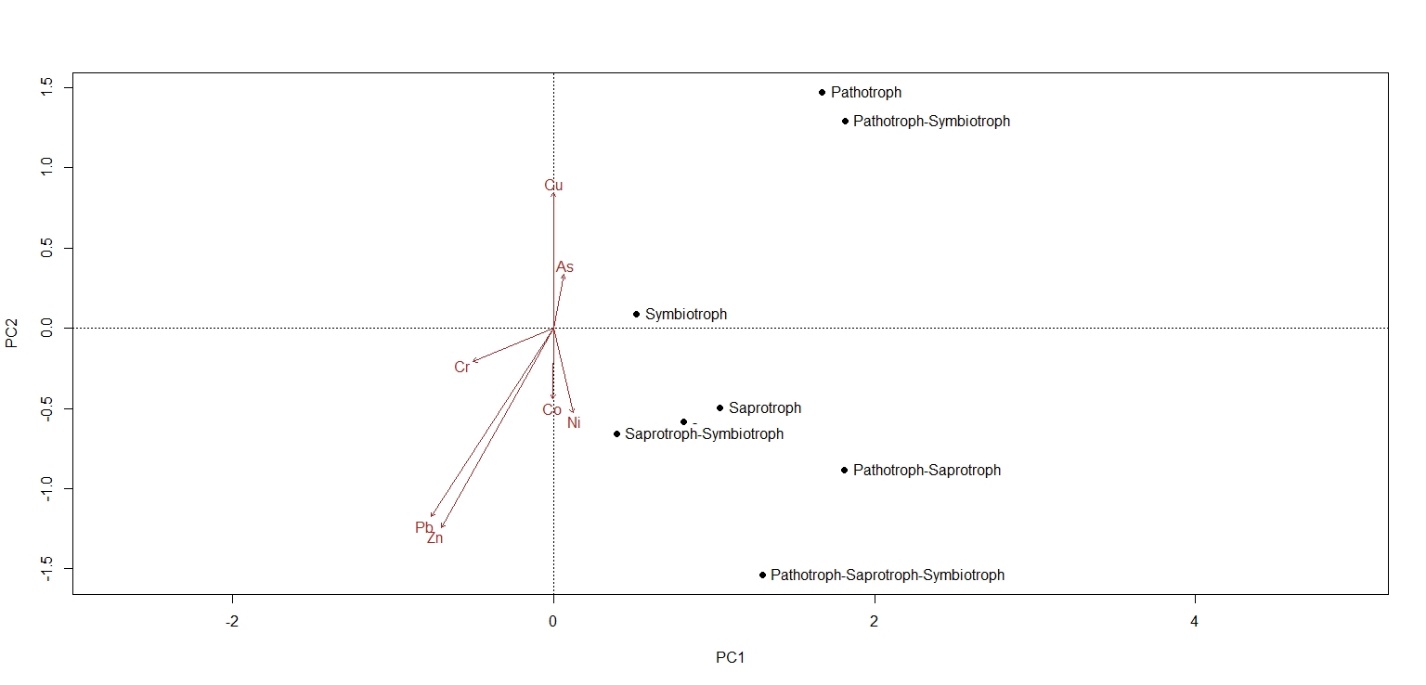
**

F2

**Figure S5**. Principal Coordinates Analyses showing ordination of dominant phylotypes with selected soil chemical parameters (1) and heavy metals concentrations (2). A: archaeal communities; B: bacterial communities; C: fungal communities at phylotype level; D: *acdS*-containing bacterial communities; E: *nifH*-containing bacterial communities; F: fungal communities classified by trophic mode. *pH: soil pH; EC: electric conductivity; OM: organic matter; P: soil phosphorus concentration; Cl; soil chloride concentration; K: soil potassium concentration; Na: soil sodium concentration; N: soil nitrogen concentration; As: arsenic; Co: cobalt; Cr: chromium; Cu: copper; Ni: nickel; Pb: lead; Zn: zinc*

**Table S1.** Primer sequences and PCR protocols used to amplify microbial DNA from soils.

| **Region of interest** | **Primers** | **Sequences** | **Initial step** | **Denaturation** | **Annealing** | **Elongation** | **Cycles** | **Final step** | **Reference** |
| --- | --- | --- | --- | --- | --- | --- | --- | --- | --- |
| **16S rRNA of Bacteria** | 341F  805R | 5’-CCTACGGGNGGCWGCAG-3’  5’GACTACHVGGGTATCTAATCC-3’ | 95 °C, 3 min | 95 °C, 30 s | 62 °C, 30 s | 72 °C, 30 s | 32 | 72 °C, 10 min | ([Wasimuddin *et al.*, 2019](#_ENREF_4)) |
| **16S rRNA of Archaea** | 340F  806rB | 5’ – CCCTAYGGGGYGCASCAG-3’  5’-GGACTACNVGGGTWTCTAAT-3’ | 95 °C, 10 min | 95 °C, 30 s | 55 °C, 30 s | 72 °C, 60 s | 30 | 72 °C, 10 min | ([Bahram *et al.*, 2019](#_ENREF_1)) |
| **ITS of Fungi** | ITS1F  ITS2 | 5’-CTTGGTCATTTAGAGGAAGTAA-3’  5’-GCTGCGTTCTTCATCGATGC-3’ | 95 °C, 2 min | 95 °C, 30 s | 55 °C, 30 s | 72 °C, 60 s | 40 | 72 °C, 10 min | ([Op De Beeck *et al.*, 2014](#_ENREF_3)) |
| ***acdS*** | acdSF5  acdSR8 | 5’-GGC AAC AAG MYS CGC AAG CT-3’  5’- CTGCACSAGSACGCACTTCA-3’ | 95 °C, 10 min | 94 °C, 30 s | 67 °C, 7 s | 72 °C, 15 s | 50 | 72 °C, 10 min | ([Bouffaud *et al.*, 2018](#_ENREF_2)) |
| ***nifH*** | PolF  PolR | 5’-TGC GAY CCS AAR GCB GAC TC-3’  5’-ATS GCC ATC ATY TCR CCG GA-3’ | 94 °C, 5 min | 94 °C, 30 s | 54 °C, 45 s | 72 °C, 45 s | 35 | 72 °C, 10 min | ([Yang *et al.*, 2019](#_ENREF_5)) |

**Table S2.** Predicted plant growth promoting activities and associated genes of bacterial communities associated with five agricultural soils collected from the Indus River Basin, Pakistan created using PICRUSt2.

| **Activity** | **Gene** | **Soil sample association** |
| --- | --- | --- |
| *Nitrogen cycling* | | |
| nitrogenase molybdenum-iron protein alpha chain | nifD | Present in all soils |
| nitrate reductase (NADH) | narG | Present in all soils |
| nitrite reductase (NAD(P)H) large subunit | nirK (large) | Choa Saidan Shah (CSS) + Daharki (D) |
| nitrite reductase (NAD(P)H) small subunit | nirK (small) | Choa Saidan Shah (CSS) + Daharki (D) |
| nitrous-oxide reductase | nosZ | Present in all soils |
| ammonia monooxygenase subunit A | amoA | Present in all soils |
| nitric oxide reductase subunit B | norB | Present in all soils |
| nitrite oxidoreductase alpha subunit | nxrA | Present in all soils |
| *Phosphorous cycling* | | |
| alkaline phosphatase | phoA, phoB | Choa Saidan Shah (CSS) |
| acid phosphatase | PHO | Present in all soils |
| 4-phytase / acid phosphatase | appA | Daharki (D) |
| acid phosphatase (class A) | phoN | Daharki (D) |
| acid phosphatase (class B) | aphA | Daharki (D) |
| *Carbon/iron cycling and stress responses* | | |
| pyrroloquinoline-quinone synthase | pqqC | Daharki (D) |
| cytochrome P450 monooxygenase | rhiH | Present in all soils |
| Fur family transcriptional regulator, ferric uptake regulator | FUR | Mirpur Mathelo (MM) |
| salicylate biosynthesis isochorismate synthase | pchA | Present in all soils |
| salicylate synthetase | mbtI, irp9, ybtS | Daharki (D) |
| isochorismate pyruvate lyase | pchB | Pakka Anna (PA) |
| indole-3-glycerol phosphate synthase | IGPS | Daharki (D) + Khewra salt mines range (KSMR) + Mirpur Mathelo (MM) + Pakka Anna (PA) |
| 1-aminocyclopropane-1-carboxylate deaminase | ACC deaminase | Choa Saidan Shah (CSS) + Daharki (D) |
